# Flavonoid compound from *Forsythia suspensa* leaves inhibits adenovirus infection related to cell cycle based on UHPLC-Q-Exactive-Orbitrap/MS and experimental validation

**DOI:** 10.3389/fcimb.2025.1627863

**Published:** 2025-08-21

**Authors:** Lingling Wang, Shuanshan Ren, Xingming Ma, Yanliu Li, Yongyong Zheng, Ling Li, Luhong Cao

**Affiliations:** ^1^ School of Comprehensive Health Management, Xihua University, Chengdu, China; ^2^ Department of Otolaryngology Head and Neck Surgery, Sichuan Provincial People’s Hospital, School of Medicine, University of Electronic Science and Technology of China, Chengdu, China

**Keywords:** UHPLC-Q-Exactive-Orbitrap/MS, *Forsythia suspensa* leaves, adenovirus, flavonoids, cell cycle

## Abstract

**Introduction:**

*Forsythia suspensa* leaves (FSL), a traditional Chinese ethnomedicinal herbal material used to prepare health-promoting infusions and pharmacologically noted for their robust anti-inflammatory, antioxidant, and broad-spectrum antiviral activities, nevertheless have an as-yet-uncharacterized molecular mechanism of action against human adenovirus (HAdV).

**Methods:**

Ultra-high-performance liquid chromatography coupled with Q-Exactive Orbitrap mass spectrometry (UHPLC-Q-Exactive-Orbitrap/MS) was employed to identification of FSL components. Publicly available GEO datasets were mined to identify HAdV-associated differentially expressed genes (DEGs). An integrated analysis of GEO datasets and network pharmacology to predict the key molecular targets of FSL flavonoids against HAdV. Ensemble molecular docking and molecular dynamics simulations assessed the stability of flavonoid–protein interactions. In vitro antiviral assays quantified FSL’s effect on HAdV replication, viral gene expression, and E1A protein levels. Flow cytometry and RT-qPCR examined cell cycle distribution and expression of cell-cycle regulators.

**Results:**

Thirty-nine active components were identified in FSL, predominantly organic acids, terpenoids, flavonoids, and lignans. An integrated analysis of GEO data revealed 990 adenovirus-associated DEGs, with network pharmacology and functional enrichment analyses further demonstrating FSL flavonoids’ preferential targeting of cell cycle regulators. *In silico* docking and simulation confirmed the stable binding of FSL-derived flavonoids to core cell cycle proteins. *In vitro*, FSL inhibited HAdV replication in a dose-dependent manner, significantly reducing viral gene transcripts and E1A protein expression. Mechanistic studies demonstrated that FSL induced G2/M phase arrest, accompanied by the downregulation of *CDC25A* expression and upregulation of *CCNB2*, *AURKA*, *CHEK1*, and *CCNA2* expression, thereby blocking progression into S-phase and impairing viral DNA synthesis.

**Discussion:**

These findings establish a pharmacological foundation for developing FSL-derived phytotherapeutics against adenoviral infections.

## Graphical Abstract

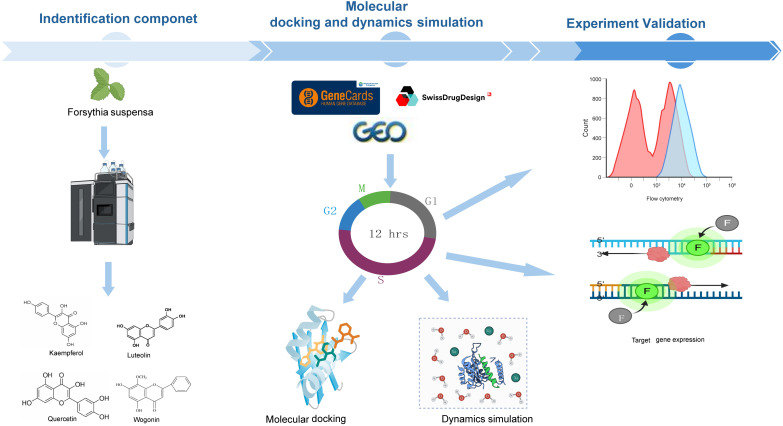

## 1 Introduction

Human adenovirus (HAdV), first isolated from adenoid by Rowe et al. in 1953 (Rowe et al., 1953), is a non-enveloped double-stranded DNA virus with seven subtypes (A–G) (Kulanayake and Tikoo, 2021). HAdV is associated with diverse clinical manifestations, including respiratory infections, conjunctivitis, and hepatitis (Lion, 2014). It contributes to 5.8%–13% of pediatric acute respiratory hospitalizations and over 50% of pneumonia cases in unvaccinated cohorts (Kajon et al., 2010). Notable transmission routes include contaminated water systems and fresh produce (Gray, 2006). Current therapeutic options are limited to nephrotoxic agents like cidofovir, which exhibits <40% efficacy against severe pneumonia (Ko et al., 2020). Notably, HAdV replication critically depends on hijacking the host cell cycle, particularly the G1/S phase transition—a mechanism validated as a promising therapeutic target (Liu et al., 2025). However, no studies have systematically explored the potential of traditional Chinese medicines (TCMs) to modulate this host-dependent viral mechanism.

Traditional Chinese medicines have a millennial history of demonstrated clinical efficacy against infectious diseases. Emerging evidence suggests that TCM exhibits a broad-spectrum antiviral activity against viruses such as influenza, SARS-CoV-2, and HAdV by simultaneously targeting multiple pathways, including the direct inhibition of viral replication. *Forsythia suspensa* (Thunb.) Vahl, one of the most widely used TCM, possesses important biological activities, such as antibacterial, antiviral, anti-inflammatory, and antioxidant activities (Jeong et al., 2021; Li W. et al., 2021; Bailly, 2022). Its leaves are integral to Lianhua Qingwen granules, a WHO-recognized nutraceutical for viral respiratory diseases including COVID-19 (Zhou et al., 2011; Liu et al., 2021; Hu et al., 2022). Modern phytochemical studies reveal FSL as a rich source of active polyphenols (e.g., phillyrin), flavonoids (e.g., kaempferol), and terpenoids (e.g., oleanolic acid), which collectively mediate the pleiotropic effects spanning antiviral (Li W. et al., 2021; Yang et al., 2022), antioxidative (Chang et al., 2008), immunomodulatory (Zeng et al., 2017), and metabolic regulatory functions (Zhang et al., 2016; Zhou et al., 2024). Notably, FSL extracts exhibit a broad-spectrum antiviral activity against COVID-19 through NF-κB pathway suppression (Ma et al., 2020) and inhibit anti-influenza viral entry via terpenoid-mediated mechanisms (Zhang et al., 2002). These properties align with the host cell cycle modulation strategy previously discussed, as NF-κB signaling and viral entry are critical determinants of G1/S phase progression during HAdV infection. However, despite its emerging role in antiviral strategies, their role in modulating host cell cycle pathways—specifically the G1/S phase dependency exploited by HAdV—remains uninvestigated.

Herein we adopted an integrative research framework spanning phytochemistry, network pharmacology, *in silico* modeling, and functional assays to systematically elucidate FSL’s anti-HAdV potential. Using UHPLC-Q-Exactive-Orbitrap/MS, we comprehensively characterized the bioactive components in FSL, encompassing organic acids, terpenoids, flavonoids, and lignans. Multi-omics network pharmacology analysis, integrated with data from healthy and HAdV-infected samples, prioritized cell cycle regulatory pathways as key targets of FSL. Through ensemble molecular docking and molecular dynamics (MD) simulations, we validated the stable binding of FSL-derived flavonoids to core cell cycle proteins, providing structural insights into their interactions. *In vitro* functional assessments demonstrated that FSL inhibited HAdV replication in a dose-dependent manner, accompanied by the downregulation of viral gene and protein expression. Notably, flow cytometry analysis was used to evaluate the cell cycle arrest induced by FSL. By elucidating FSL’s capacity to block HAdV replication through cell cycle process modulation, this study positions FSL as a sustainable, multi-target ingredient to mitigate viral risks in immunocompromised patients.

## 2 Methods

### 2.1 Detection active components of *Forsythia suspensa* leaves by UPLC/Q-TOF MS analysis

*Forsythia suspensa* leaves were purchased from Chengdu Pi Du Tianyitang Traditional Chinese Medicine Clinic Co., Ltd. The identity of the herbs as genuine was confirmed by Professor Jianhua Huang, Chengdu University of Traditional Chinese Medicine. The FSL leaves were dissolved in ethanol to prepare a sample according to Zhou et al. (2021) and were concentrated to 1/3 volume using a vacuum rotatory evaporator at 90°C. This extraction was repeated five times, then the extract was filtered to obtain the test solution. All stock solutions were stored at 4°C until use. The analysis of FSL was carried out by using a Thermo Hypersil GOLD C18 column (100 mm × 2.1 mm i.d., 1.9 μm) maintained at a temperature of 30°C. The mobile phase was comprised of acetonitrile (A) and an aqueous solution of 0.1% formic acid (B). The gradient elution profile was as follows: from 0 to 5 min, the composition changed from 30% to 65% A; from 5 to 16 min, it increased from 65% to 80% A; then from 16 to 25 min, it rose from 80% to 90% A; and finally, from 25 to 30 min, it transitioned completely to 100% A. The flow rate was established at a rate of 0.3 mL/min, with an injection volume set at 5 μL. High-resolution mass spectrometry detection utilized a Q-Exactive Orbitrap MS system (Thermo Scientific, Waltham, MA, USA), which featured a heated electrospray ionization source operating in both positive (ESI+) and negative (ESI−) ion modes. Data acquisition occurred within the m/z range of 100–1,500 Da under full MS scan conditions. For ESI-MS analysis, specific parameters were configured: the spray voltage was adjusted to 3,000 V for positive ions and 3,500 V for negative ions; the sheath gas flow and auxiliary gas flow rates were set at 35 and 10 arbitrary units, respectively; the capillary temperature remained constant at 320°C, while the auxiliary gas heater temperature was kept at 350°C; stepped normalized collision energies were defined as 20, 40, and 60 eV; and scanning mode operated in full MS/dd-MS2 with resolutions recorded at 70,000 FWHM for full MS scans and 17,500 FWHM for dd-MS2 scans. Data collection and processing utilized Xcalibur version 4.1 along with Compound Discover version 3.0 software tools.

### 2.2 Screening of the targets of *Forsythia suspensa*

The Simplified Molecular Input Line Entry System (SMILES) formulas of the active components were then entered into the Swiss Target Prediction database (http://www.swisstargetprediction.ch/) to identify their target proteins. Subsequently, the target names were standardized using the UniProt database (https://www.uniprot.org/). The GeneCards database (https://www.genecards.org/) was also screened for targets related to the active ingredients. Venn diagrams were created to visualize the intersection of FSL target genes with the target of HAdV infection.

### 2.3 The target of HAdV infection analysis

The mRNA dataset GSE68004 from the GEO database was downloaded, and it contained 37 healthy individuals and 19 HAdV-infected patients (Jaggi et al., 2018). The “limma” package in R4.3.3 software was according to the standard of log2FC >1 and *P <*0.05. Differentially expressed genes between healthy individuals and HAdV-infected patients were screened out.

### 2.4 Construction of drug-component-target–KEGG pathway network and protein–protein interaction analysis

The STRING database (https://cn.string-db.org/) constructed the PPI network, and the tsv file was downloaded. Cytoscape was used to construct the “drug-ingredient–target–KEGG pathway” network. The CytoNCA plug-in of Cytoscape software was used to analyze the degree centrality (DC), etc., and it was made as an index for screening and predicting therapeutic targets to topological analyses.

### 2.5 Gene function enrichment analysis

Predicted therapeutic targets were subjected to functional annotation and functional enrichment analysis to investigate the biological significance of the genes or targets. DAVID (https://david.ncifcrf.gov/) database was performed to screen and draw biological processes and signaling pathways.

### 2.6 Molecular docking simulation

The 3D structure of flavonoids components from FSL was obtained through the RCSB database (https://www.rcsb.org/). The protein structure was dehydrated, hydrogenated, and charged by Pymol, and then Autodock Vina was used to simulate the molecular docking of the active ingredient of FSL and the core target. The binding strength of the two was evaluated by binding energy value, and the results were visualized by Pymol.

### 2.7 Molecular dynamics simulation analysis

MD simulations were performed using Gromacs 2022.3 with the Amber99sb-ildn force field. Small molecules were parameterized with GAFF via AmberTools22. After energy minimization and NVT/NPT equilibration (100 ps each), production runs were conducted for 100 ns with a 2-fs timestep at 300 K. Additionally, hydrogen atoms were introduced, and the RESP potential was computed using Gaussian 16W software. This potential data was subsequently integrated into the MD system’s topology file. During the simulations, a constant temperature of 300 K and an atmospheric pressure of 1 bar were maintained. The Amber99sb-ildn force field was employed, with Tip3p water molecules acting as the solvent. To maintain system charge neutrality, an adequate number of Na^+^ ions were introduced. Energy minimization was achieved through the steepest descent approach. The system was then equilibrated under isothermal isovolumic (NVT) and isothermal isobaric (NPT) conditions for 100,000 steps each, with a coupling constant of 0.1 ps over a period of 100 ps. Following equilibration, a free MD simulation was conducted, comprising 5,000,000 steps at a step length of 2 fs, totaling 100 ns. The results of the MD simulations, including RMSD, RMSF, Rg value, SASA, and H-bonds, were visualized using GraphPad Prism Version 9.5. The Gibbs free energy was calculated from RMSD and Rg values using a custom “bash” script and was visualized in both 3D and 2D landscapes through Origin (2021) software.

### 2.8 Cytotoxicity test

A549 cells at a density of 4–6 × 10^4^ cells/mL were used to inoculate two 24-well plates. On the next day, the cells were grown to be a monolayer of cells with a density of approximately 90%. A total of 10 concentrations were obtained by twofold ratio for serial dilution in the cell culture medium (DMEM + 10% FBS) with cell maintenance liquid. The waste liquid was discarded out of the 24-well plates, and they were washed gently with PBS solution for three times. Furthermore, 1 ml of each concentration gradient drug (drug diluent water) was added to the 24-well plates, and each same-concentration gradient drug was added to four wells. Four wells of normal cells and four wells of 5% concentration of PBS were set up as control groups. They were next cultured under the condition of 5% CO_2_ and 37°C for 96 h and observed for any cell changes. The normal cell group and 5% DMSO group served as a control standard, and cytopathic method was used to detect the cellular minimal effective dose of various experimental drugs.

### 2.9 Real-time PCR

We tested the FSL concentrations (150–750 μg/mL) via CCK-8 assay and confirmed >90% cell viability in all samples. We test the viral particles according to Li et al (Li X. et al., 2021). Briefly, the A549 cell monolayers in 24-well plates were infected with 100 TCID_50_ of HAdV-2 for 1 h at 37°C. Then, the monolayers were washed twice with MEM and incubated for 7 days. The infected cells were then observed daily under microscopy to check the cytopathic effect (CPE). So, we used different concentrations (containing 0, 150, 250, and 750 μg/mL) to test the virus gene and protein. After A549 cells were infected with HAdV 2 (MOI = 1) for 2 h, they were treated with FSL of different concentrations (containing 0, 150, 250, and 750 μg/mL) for 24 h. HAdV2 was a gift from professor Erguang Li. Subsequently, RNA was isolated from these cells using the RNA isolator Total RNA extraction reagent (Vazyme, R401-01-AA). Then, the RNA was extracted with chloroform. The supernatant fluid was mixed with the same volume of isopropanol to extract the RNA. Finally, the RNA was washed three times with anhydrous ethanol and dissolved in DNase-free water. The RNA concentration of each sample was measured using Nano800 Plus (Jiapeng, Shanghai, China). Then, 1 µg of RNA from each sample was reverse-transcribed to DNA templates with a HiScript II Q RT SuperMix for qPCR (+gDNA wiper) kit (Vazyme, R223-01). The cDNA template was double-diluted with RNase water. The qPCR master mix was followed according to the manufacturer’s instructions (Vazyme, Q712-02). The primers used were designed as presented in **Table 1**. The results were processed using Archimed X6 (ROCGENE) and analyzed. This experiment was conducted in duplicate and repeated three times.

**Table 1:** Primer sequences used in the experiments.

Primer name	Sequences (5′-)	Size (bp)	Location	GenBank no.
E1F	TGCAAGTGTGGCGGAACA	159	nt126-284	AC_000008.1
E1R	TCCCGCGAAAATGGCCAA			
E2F	TGCAAGCCATCAACAAAGCC	244	nt26048-26291	AC_000008.1
E2R	CCTCCTCCTCGTCCAAAACC			
E3F	ACCACTGCTACCGGACTAAC	90	nt29510-29599	J01917.1
E3R	AAACCACCACATGTCCAAGC			
E4F E4R	CGGTAAACACATCAGGTTGGT GCTGTAATGTTGTCTCTACGCC	100	nt35304-35403	J01917.1
GAPDHF	ACAACTTTGGTATCGTGGAAGG	101	nt519-540	NM_001357943.2
GAPDHR	GCCATCACGCCACAGTTTC		nt619-610	
AURKA F	CTTGTCAACCGTGTACCAGC	106	nt56390172-56390191	NC_000020.11
AURKA R	CACCTGGGGGAAAAGCGTAA		nt56390277-56390258	
CCNB2 F	TGCTCTGCAAAATCGAGGACA	180	nt461-481	NM_004701.4
CCNB2 R	GCCAATCCACTAGGATGGCA		nt640-621	
CDC25A F	GTGAAGGCGCTATTTGGCG	108	nt549-567	NM_001789.3
CDC25A R	TGGTTGCTCATAATCACTGCC		nt656-636	
CHEK1 F	ATATGAAGCGTGCCGTAGACT	183	nt992-1012	NM_001114122.3
CHEK1 R	TGCCTATGTCTGGCTCTATTCTG		nt1174-1152	
CCNA2 F	GGATGGTAGTTTTGAGTCACCAC	202	nt699-721	NM_001237.5
CCNA2 R	CACGAGGATAGCTCTCATACTGT		nt900-878	

### 2.10 Western blot

Seeding of A549 5 × 10^5^ cells/well in was carried out on a six-well plate, and the cells were incubated under the condition of 5% CO_2_ and 37°C for 24 h. The next day, we treated with different concentration of FSL extract (150-750 μg/mL) after infection with HAdV2 (MOI=1). Then, the cell medium was discarded, and the cells were washed twice with PBS. Then, the cells were lysed with NP-40 lysis buffer (Beyotime, P0013F) on ice to obtain the protein, and the protein concentration of each well was measured by the Bradford method. Furthermore, 20 μg protein of each sample was denatured by using 5× SDS-PAGE sample loading buffer (Beyotime, P0015). Then, the denatured proteins were separated using 10% SDS-PAGE gel electrophoresis (Beyotime, P0012AC) and transferred to polyvinylidene fluoride (PVDF) membranes (Millipore, USA). The membranes were blocked by using 5% skimmed milk and incubated with Adenovirus-2/5 E1A antibody (1:1,000, Santa Cruz, sc-25, lot #K1022), CDC25A (1:1,000, Beyotime, AF6459), CCNB2 (1:1,000, zenbio, R381384), AURKA (1:1,000, Zenbio, 222073), CHEK1 (1:1,000, Beyotime, AF6498), and CCNA2 (1:1,000, Beyotime, AF6624), with GAPDH (1:2,000, Beyotime, AF0006) serving as the loading control. Then, the membranes were incubated with the HRP-labeled goat anti-mouse secondary antibody (1:2,000, Beyotime, A0216) or HRP-labeled goat anti-rabbit secondary antibody (1:2,000, Beyotime, A0208), and the images were exposed by using Tanon 5200 (Tanon, Shanghai, China).

### 2.11 Cell cycle analysis

Following digestion, quenching, and centrifugation, the A549 cells (1 × 10^6^ cells/mL) were resuspended in cell culture medium and seeded into a six-well culture plate for 24 h. For cell cycle analysis, the cells were pretreated with FSL at concentrations ranging from 150 to 750 µg/mL, followed by HAdV infection at an MOI of 1. After infection, the cells were incubated for 24 h before being harvested for analysis. The harvested cell suspensions were subjected to dual PBS washing cycles (4°C, 1,000 ×*g*, 10 min). These were fixed via dropwise addition of 4.5 mL ice-cold 70% ethanol during vortex mixing, followed by 18 h of fixation at -20°C. The pelleted cells underwent PBS rehydration (2 mL, 1,000 ×*g*, 10 min) and RNA digestion with 500 μL RNase A (100 μg/mL, 37°C, 30 min, dark). Nuclear staining was carried out using PI solution (25 μL of 1 mg/mL stock, 10 min, dark). The cell cycle profiles were ultimately quantified using a LongCyte™3 flow cytometer (Challenbio, Beijing, China) with FlowJo_v10.8.1 analysis software.

### 2.12 Statistical analysis

All data were presented as mean ± SEM from three independent trials. Statistical evaluation was conducted via one-way analysis of variance (ANOVA) with Tukey’s *post-hoc* test analysis using GraphPad Prism version 9.5.0.

## 3 Results

### 3.1 Identifying and screening of active components and targets in FSL

**Figure 1:**
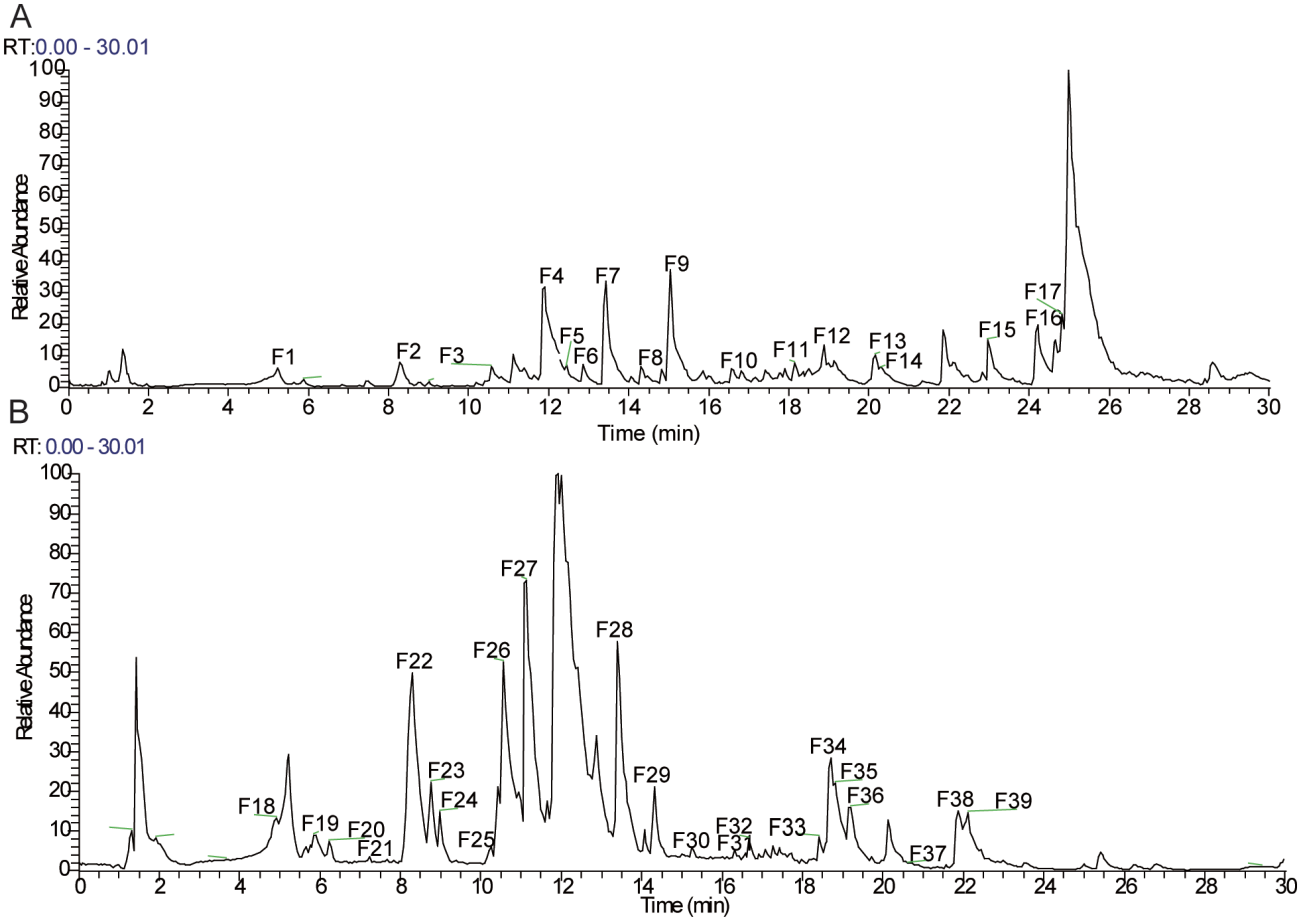
Total ion chromatograms (TICs) of *Forsythia suspensa* leaf (FSL) extract acquired by UHPLC-Q-Exactive-Orbitrap/MS. **(A)** Total ion chromatogram acquired in positive ionization mode (ESI+). **(B)** Total ion chromatogram acquired in negative ionization mode (ESI−).

The total ion chromatogram (TIC) in both positive and negative ion modes is displayed in **Figure 1**. The structures of the compounds were confirmed by comparing their retention times, accurate and high-resolution masses, as well as MS/MS fragments with standards or related literature. A total of 39 components were identified (**Table 2**; **Figure 1**), comprising 11 organic acids, four terpenoids, two lignans, four flavonoids, and 18 other substances.

**Table 2:** Components of FSL by UHPLC-Q-Exactive-Orbitrap/MS.

No.	RT (min)	Extracting ions	Measured mass (m/z)	Error (ppm)	Formula	Main secondary fragment ions (MS/MS) and sources	Identity	Category	Case no.
F1	5.32	[M+NH4]^+1^	152.057	17.026	C_5_H_5_N_5_O	110 124 135	Guanine	Other	73-40-5
F2	8.85	[M+H]^+1^	260.202	0.001	C_17_H_25_NO	159 172 228	4′-Methyl-α-pyrrolidinohexanophenone	Other	1391052-36-0
F3	10.45	[M+H]^+1^	373.184	0.000	C_21_H_2_O_2_H_3_NO_5_	193 203 253	Heroin-d3	Other	219533-68-3
F4	11.89	[M+H]^+1^	135.044	0.000	C_8_H_6_O_2_	89 107 117	Phthaldialdehyde	Organic acids	643-79-8
F5	12.45	[M+H]^+1^	287.056	0.001	C_15_H_10_O_6_	107 135 153	Kaempferol	Flavonoids	520-18-3
F6	12.86	[M+H]^+1^	303.050	0.000	C_15_H_10_O_7_	137 153 229	Quercetin	Flavonoids	117-39-5
F7	13.42	[M+H+MeOH]^+1^	189.091	32.023	C_8_H_8_N_6_	158 159 174	6-(3-Pyridyl)-1,3,5-triazine-2, 4-diamine	Other	18020-61-6
F8	14.45	[M+H-H2O]^+1^	269.045	0.003	C_15_H_11_ClN_2_O_2_	84 189 241	Oxazepam	Other	604-75-1
F9	15.00	[M+H]^+1^	293.211	0.000	C_18_H_28_O_3_	215 257 275	12-oxo Phytodienoic acid	Organic acids	85551-10-6
F10	16.58	[M+H]^+1^	285.076	0.000	C_16_H_12_O_5_	168 179 270	Wogonin	Flavonoids	632-85-9
F11	18.15	[M+Na]^+1^	365.136	0.001	C_20_H_22_O_5_	105 163 343	Dipropyleneglycol dibenzoate	Other	20109-39-1
F12	18.83	[M+H]^+1^	403.246	0.002	C_24_H_34_O_5_	343 357 393	Dehydrocholic acid	Other	81-23-2
F13	19.99	[M+H]^+1^	471.348	0.001	C_30_ H_46_ O_4_	95 107 189	18-β-Glycyrrhetinic acid	Terpenoid	471-53-4
F14	20.32	[M+H+MeOH]^+1^	317.248	32.026	C_21_H_32_O_2_	95 109 257	Eicosapentaenoic acid methyl ester	Organic acids	2734-47-6
F15	22.84	[M+H]^+1^	427.394	0.001	C_30_H_50_O	95 107 109	Lupeol	Terpenoid	545-47-1
F16	24.29	[M+Na]^+1^	465.371	0.001	C_30_H_50_O_2_	81 95 109	Betulin	Terpenoid	473-98-3
F17	24.85	[M+H]^+1^	535.271	0.001	C_33_H_34_N_4_O_3_	435 447 507	SB236057A	Other	180084-01-9
F18	3.60	[M-H]^-1^	243.062	0.000	C_9_H_12_N_2_O_6_	110 146 174	Uridine	Organic acids	611-73-4
F19	5.79	[M-H]^-1^	129.018	0.001	C_5_H_6_O_4_	85 128 129	Citraconic acid	Organic acids	498-23-7
F20	6.13	[M-H]^-1^	131.033	0.001	C_5_H_8_O_4_	87 102 115	Methylsuccinic acid	Organic acids	498-21-5
F21	7.32	[M-H]^-1^	153.018	0.001	C_7_H_6_O_4_	109 110 123	Gentisic acid	Organic acids	490-79-9
F22	8.42	[M-H]^-1^	137.023	0.001	C_7_H_6_O_3_	81 92 109	2,5-Dihydroxybenzaldehyde	Other	1194-98-5
F23	8.76	[M-H]^-1^	461.167	0.001	C_20_H_30_O_12_	59 73 135	Bioside	Other	12634-05-8
F24	8.98	[M-H]^-1^	149.023	0.001	C_8_H_6_O_3_	95 105 121	Phenylglyoxylic acid	Organic acids	611-73-4
F25	10.20	[M+FA-H]^-1^	179.034	46.006	C_9_H_8_O_4_	134 145 179	Caffeic acid	Organic acids	331-39-5
F26	10.64	[M-H]^-1^	163.039	0.001	C_9_H_8_O_3_	107 134 135	3-Coumaric acid	Organic acids	588-30-7
F27	11.19	[M-H]^-1^	567.209	0.000	C_26_H_34_O_11_	160 175 329	Lariciresinol 4-O-glucoside	Lignans	143663-00-7
F28	13.25	[M-H]^-1^	149.060	0.001	C_9_H_10_O_2_	121 147 148	Hydrocinnamic acid	Organic acids	501-52-0
F29	14.29	[M-H+HAc]^-1^	357.135	60.021	C_20_H_22_O_6_	83 151 163	Matairesinol	Lignans	580-72-3
F30	15.34	[M-H]^-1^	351.218	0.000	C_20_H_32_O_5_	59 289 307	Lipoxin B4	Other	98049-69-5
F31	15.47	[M-H]^-1^	285.041	0.001	C_15_H_10_O_6_	107 151 239	Luteolin	Flavonoids	491-70-3
F32	16.62	[M-H]^-1^	351.218	18.010	C_20_H_34_O_6_	59 289 307	15(R),19(R)-hydroxy Prostaglandin F2?	Other	64625-53-2
F33	18.29	[M-H]^-1^	487.343	0.001	C_30_H_48_O_5_	125 379 409	Asiatic acid	Terpenoid	464-92-6
F34	18.70	[M-H]^-1^	333.207	18.010	C_20_H_32_O_5_	273 289 315	Prostaglandin F3?	Other	745-64-2
F35	18.86	[M-H]^-1^	293.213	0.001	C_18_H_30_O_3_	183 235 275	13(S)-HOTrE	Other	87984-82-5
F36	19.35	[M-H]^-1^	345.208	0.000	C_21_H_30_O_4_	81 113 269	Corticosterone	Other	50-22-6
F37	20.73	[M-H]^-1^	435.255	0.003	C_21_H_41_O_7_P	214 215 228	Oleoyl-L-α-lysophosphatidic acid	Other	65528-98-5
F38	21.85	[M-H^-1^	349.202	18.010	C_20_H_32_O_6_	287 305 331	11-dehydro Thromboxane B2	Other	67910-12-7
F39	22.07	[M-H]^-1^	279.233	0.000	C_18_H_32_O_2_	50 91 96	Linoleic acid	Other	60-33-3

To convert the target protein names into gene names, the UniProt database was utilized. After removing duplicates, a total of 556 corresponding gene targets were obtained.

### 3.2 HAdV infection-related targets

**Figure 2:**
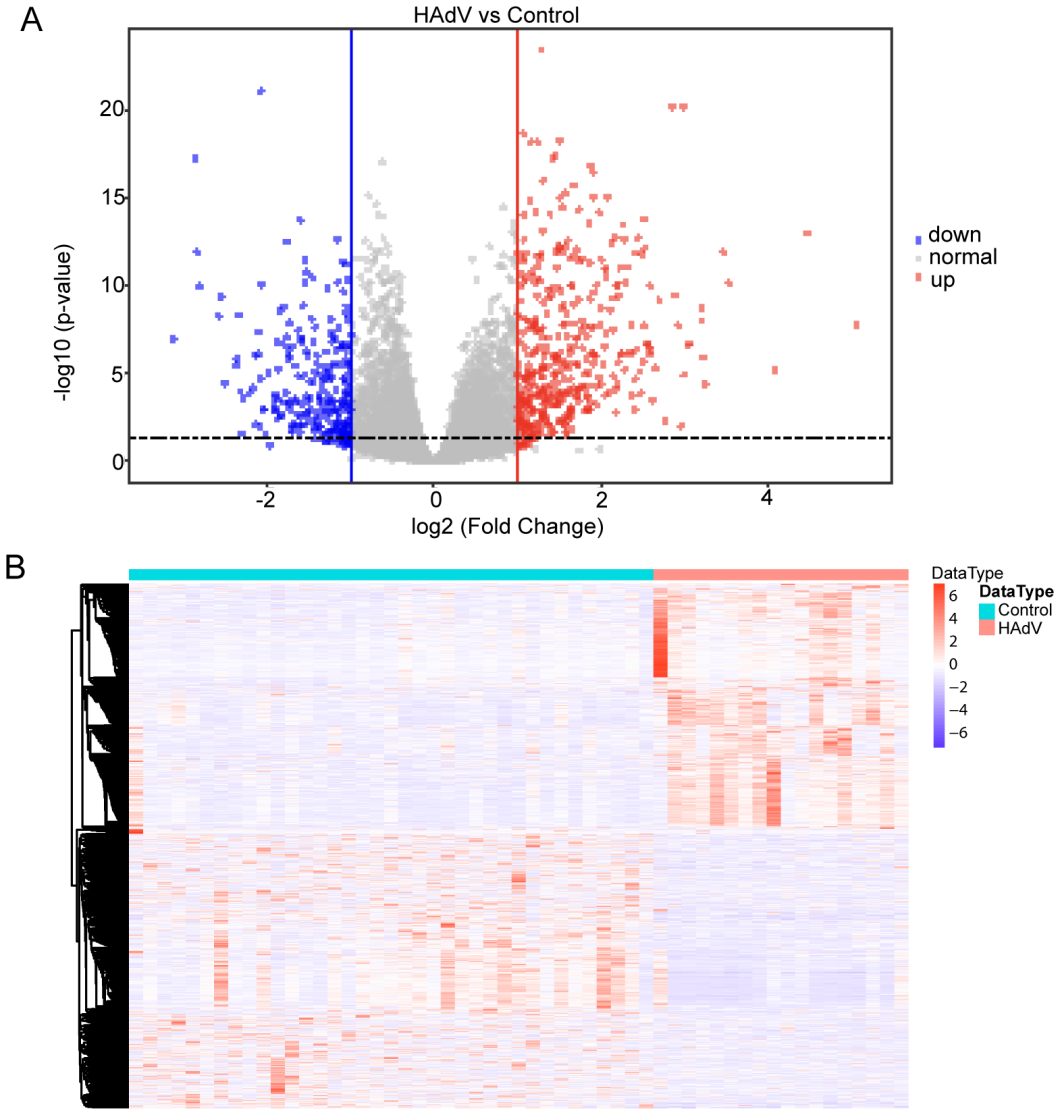
Transcriptomic profiling of HAdV infection targets using GEO dataset GSE68004. **(A)** Volcano plot of differentially expressed genes (DEGs) between HAdV-infected patients (*n* = 19) and healthy controls (*n* = 37). Significantly dysregulated genes (|log_2_FC| > 1; adjusted *p* < 0.05) are highlighted. Significantly upregulated genes are shown in red, downregulated genes in blue, and non-significant genes in gray. **(B)** Heatmap of differentially expressed genes (DEGs) across all samples. Expression values are shown as Z-scores (rows), with hierarchical clustering of both genes and samples to illustrate expression patterns in HAdV-infected versus control conditions.

A total of 37 normal and 19 HAdV-infected peripheral blood RNA-seq samples were obtained from the GEO database. By employing DESeq2 and setting a significance threshold at a *P*-value of less than 0.05 along with an absolute log2 fold change greater than 1, we detected 990 genes that were differentially expressed (DEGs) when comparing the normal group to the HAdV-infected group. To illustrate the data, a volcano plot was created, highlighting 467 genes that were upregulated and 523 genes that were downregulated (**Figure 2A**). Additionally, a heatmap was generated to depict the expression patterns of these DEGs (**Figure 2B**), and the top 10 upregulated and top 10 downregulated DEGs are presented in **Table 3**. The 990 DEGs were selected as potential targets of HAdV infection and were further analyzed in subsequent studies.

**Table 3:** Top 10 upregulated and top 10 downregulated differentially expressed genes from RNA-Seq analysis.

Series number	Gene symbol	Log2 fold change	Adjusted *P*-value
1	CD177	5.079935285	1.90E-08
2	PBK	4.490752631	1.04E-13
3	CDC25A	4.094814715	6.37E-06
4	C1QB	3.552482121	7.73E-11
5	TIMD4	3.483735656	1.33E-12
6	OTOF	3.260289963	4.21E-05
7	GALNT14	3.233692778	1.20E-06
8	HP	3.217964449	1.77E-09
9	LOC100130904	3.214324595	9.64E-09
10	CDCA3	3.074149311	1.84E-07
11	GPR44	-3.132832741	1.09E-07
12	NOV	-2.876359812	5.17E-18
13	TTC25	-2.845657218	1.32E-12
14	PDZK1IP1	-2.824014479	1.08E-10
15	HEMGN	-2.593096177	5.46E-09
16	BOAT	-2.557107767	4.27E-10
17	SMPD3	-2.511031938	3.51E-05
18	TGM2	-2.393505426	3.53E-06
19	VWCE	-2.373379425	1.41E-06
20	LOC392382	-2.339248535	4.86E-09

### 3.3 The therapeutic targets of FSL against HAdV infection and PPI network analysis

**Figure 3:**
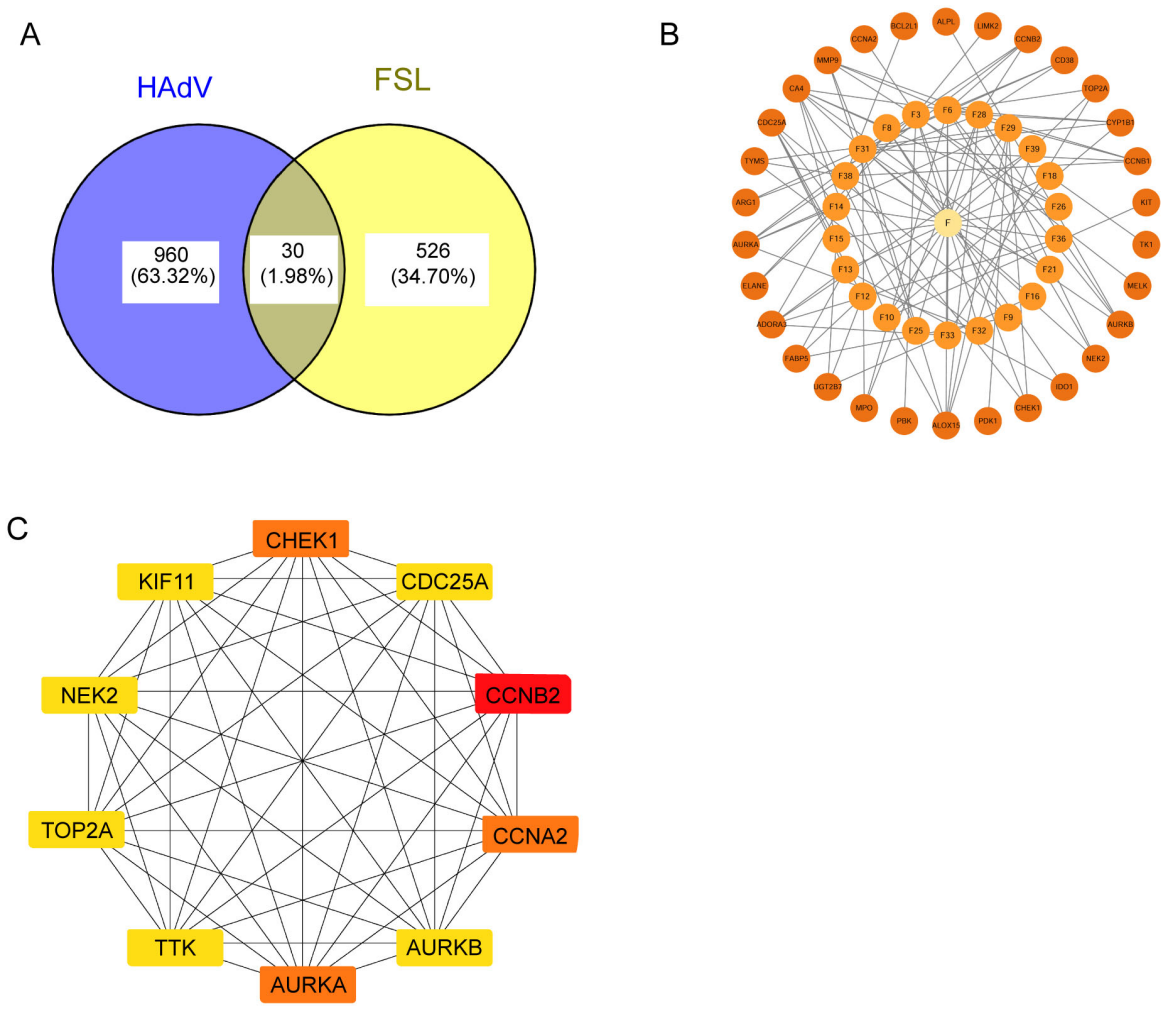
Identification of FSL therapeutic targets for HAdV infection. **(A)** Venn diagram of shared targets between FSL bioactive components (556 targets) and HAdV infection-associated differentially expressed genes (990 DEGs), identifying 30 common targets (intersection). **(B)** A compound–target interaction network for the 30 overlapping proteins. The central yellow node (“F”) represents FSL’ s components; smaller orange nodes represent the target proteins. **(C)** The protein–protein interaction (PPI) network constructed from the 30 overlapping targets was analyzed using the MCC algorithm to identify the top 10 hub proteins with the highest interaction scores. The network was visualized in Cytoscape, where the node color ranges from light yellow to red, indicating increasing degrees of importance.

To get the therapeutic targets of FSL against HAdV infection, we made the intersection of disease targets and component target. A total of 990 disease targets were matched with 556 drug targets, resulting in 30 common targets that were selected for further investigation (**Figure 3A**). The “drug ingredient–targets” network was constructed by using Cytoscape (**Figure 3B**). By utilizing the STRING database, interactions between these targets were constructed and then visualized by using Cytoscape. The resulting network diagram displayed 27 nodes and 96 edges, with nodes of higher degree values appearing with darker colors and larger sizes at the center of the protein–protein interaction (PPI) network (**Figure 3C**). The core genes contained *CCNA2*, *CHEK1*, and *AURKA* (degree was 14 throughout), *CDC25A* (degree = 13), *CCNB2*, *KIF11*, *TOP2A*, *NELK*, *AURKB*, *PBK*, and *TYMS* (degree was 12 throughout), as well as other *PLK4* and *TTK* (degree was both 11) of FSL for anti-HAdV infection.

### 3.4 Gene function enrichment analysis

**Figure 4:**
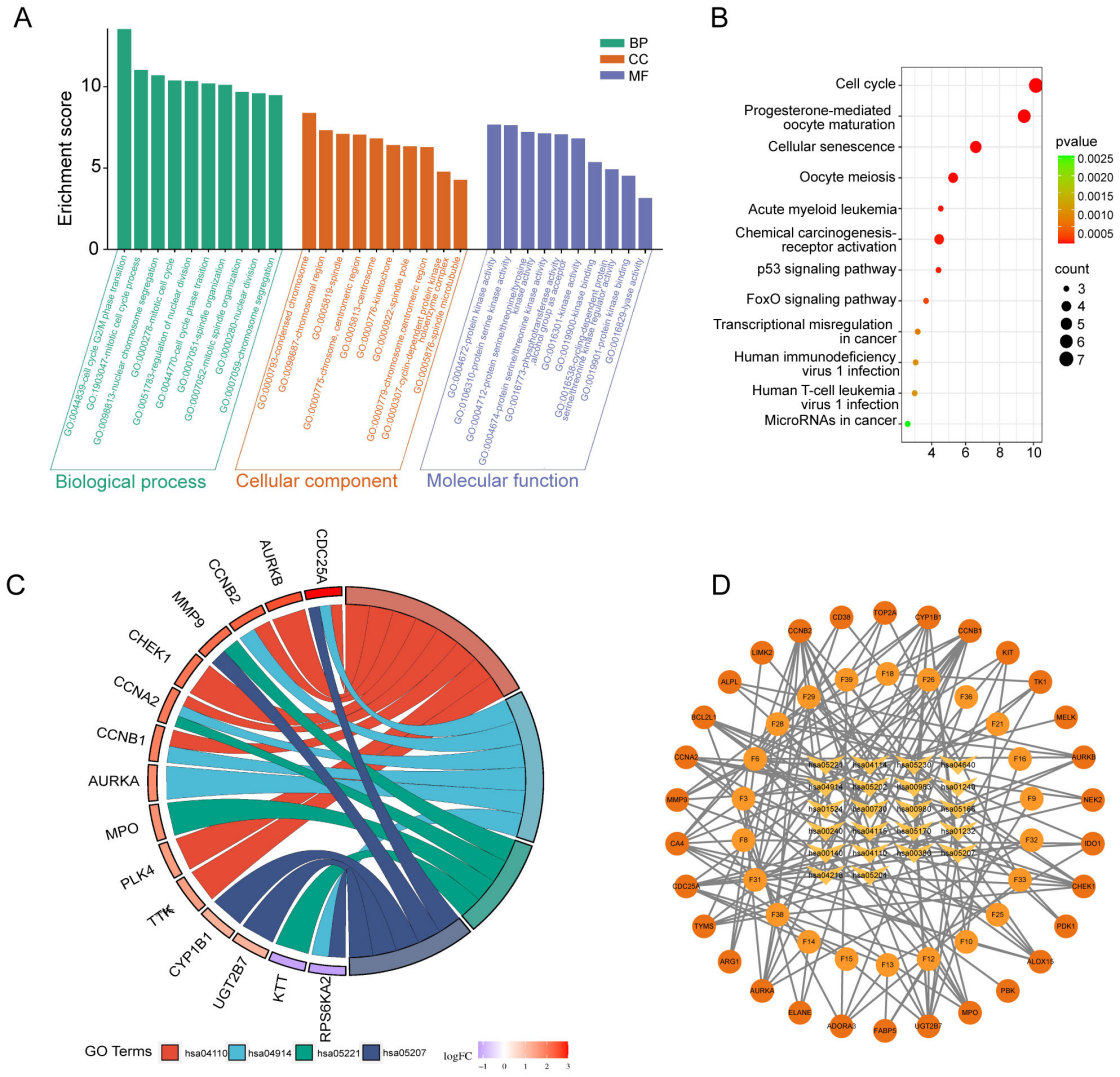
KEGG and GO enrichment analysis of FSL therapeutic targets against HAdV infection. **(A)** Bar chart of significantly enriched terms (FDR < 0.05) across biological processes (BP), cellular components (CC), and molecular functions (MF) obtained from the GO enrichment analysis. The bar height indicates enrichment significance (−log_10_ (P)); the color denotes category (BP: blue; CC: green; MF: red). **(B)** KEGG pathway bubble chart of the top 20 enriched pathways. The X-axis shows gene ratios, the Y-axis lists pathway names. Bubble size reflects gene count, and color intensity corresponds to –log_10_ (*p*-value). **(C)** Sankey diagram illustrating connections between 30 FSL-HAdV shared targets (left) and enriched KEGG pathways (right). Line width correlates with gene count per pathway. **(D)** Compound–target–pathway interaction network. Nodes represent FSL active compounds, therapeutic targets, and KEGG pathways; edges denote documented interactions. Node color and shape (see inset) distinguish compounds, targets, and pathways, illustrating the multi-level mechanism of FSL against HAdV.

The analysis of GO enrichment for 30 key genes was conducted using “R,” with a significance threshold established at *P <*0.01. The biological process (BP) enrichments were mainly cell cycle G2/M phase transition, mitotic cell cycle process, nuclear chromosome segregation, mitotic cell cycle, nuclear division regulation, cell cycle phase transition, spindle tissue, etc. Cellular component (CC) enrichments were mainly intracellular components related to the cell cycle. Molecular function (MF) enrichment was involved in various protein kinase and lyase activities (**Figure 4A**). The KEGG signaling pathway, the target gene of FSL against HAdV infection, was involved in a total of 12 pathways, including cell cycle, progesterone-mediated oocyte maturation, acute leukemia, chemocarcinogenicity-receptor activation, human immunodeficiency virus type 1 infection, and human T-cell leukemia virus type 1 infection. The heatmap shows the top 10 pathway (**Figure 4B**). A chord diagram was generated to illustrate the connections between target genes and enriched pathways (**Figure 4C**). The KEGG pathway–key gene–active ingredient relationship network was constructed by using Cytoscape (**Figure 4D**). The core genes *CCNA2*, *AURKA*, *CHEK1*, *CCNB2*, and *CDC25A* were involved in five or more virus-related signaling pathways.

### 3.5 Molecular docking results

**Figure 5:**
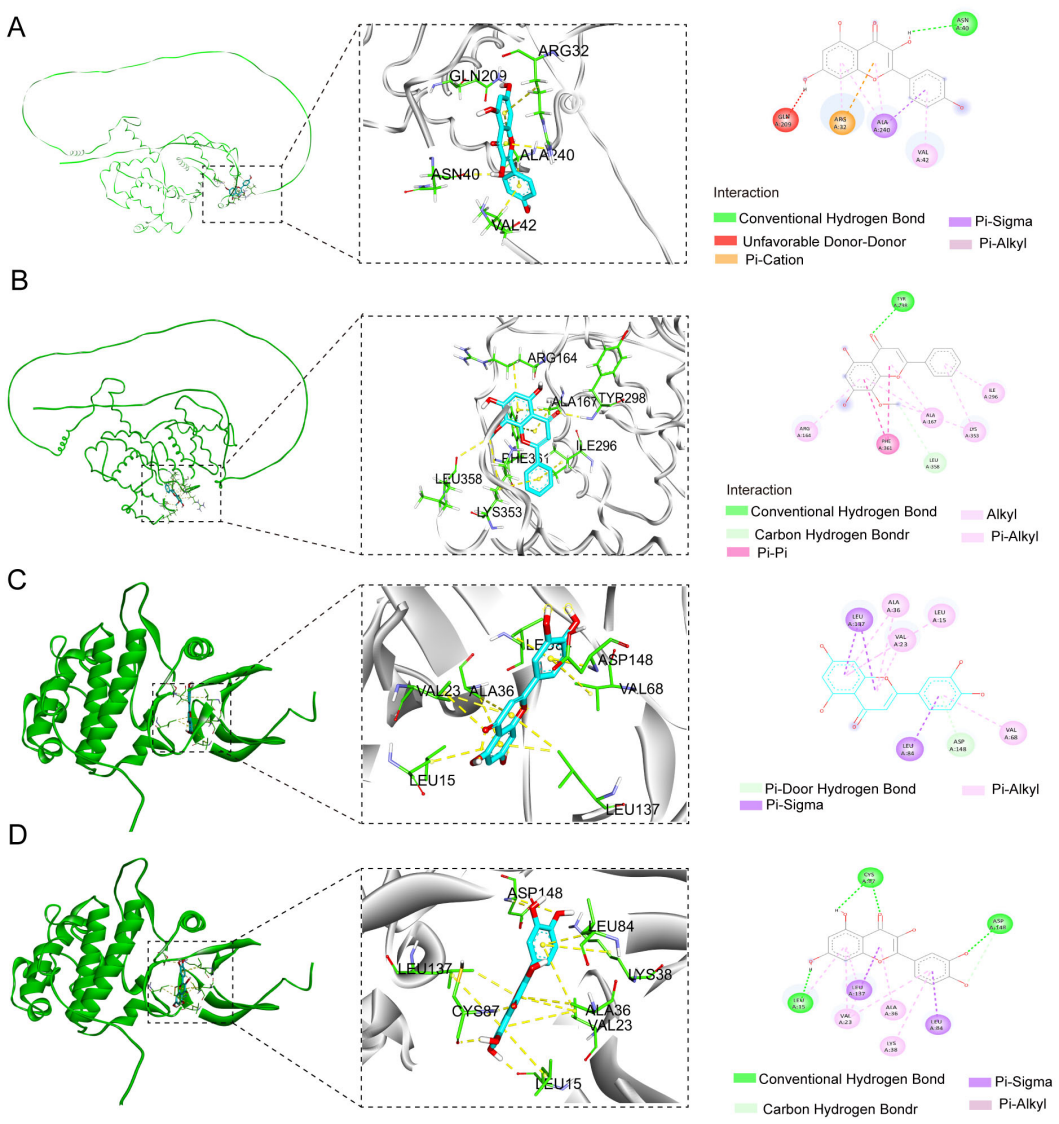
Molecular docking configurations of FSL-derived flavonoids with core cell cycle regulators. Yellow dashed lines denote hydrogen bonds; light blue spokes indicate hydrophobic contacts. All poses were obtained via ensemble docking with AutoDock Vina and visualized in Discovery studio. **(A)** Kaempferol–CCNB2 complex, **(B)** wogonin–CCNB2 complex, **(C)** luteolin–CHEK1 complex, and **(D)** quercetin–CHEK1 complex.

In order to validate the findings that four flavonoids’ components bind with their key targets, molecular docking techniques were used to assess it. The spatial coordinates of the docking configurations and comprehensive details relating to the targets and compounds used in the analysis can be found in **Table 4**. To provide a visual depiction of the interactions between compounds and targets, the binding modes were illustrated using PyMOL-1.7.2.1 and Discovery Studio 2020, with the results showcased in **Figures 5A–D**. The binding energy outcomes from the docking simulations varied, ranging from -6.7 to -9.2 kcal/mol, which suggests that these molecular complexes exhibit stable binding characteristics, indicating promising interactions between the compounds and the selected targets. Additionally, we observed that kaempferol, luteolin, and quercetin exhibit a strong binding activity with *CHEK1* and *CCNB2* (**Figures 5A–D**). Therefore, these compounds are considered candidate drug molecules.

**Table 4:** Virtual dock of the four flavonoid compounds from FSL for the hub genes.

Gene symbol	Kaempferol	Luteolin	Quercetin	Wogonin
CCNA2	6.7	-6.4	-6.7	-6.3
AURKA	-6.2	-6.7	-6.2	-6.4
CCNB2	-8.3	-8.1	-8.1	-8.7
CDC25A	-6.8	-7.0	-6.9	-6.6
CHEK1	-8.1	-9.2	-9.2	-7.9

### 3.6 Molecular dynamics simulation analysis

Furthermore, a 100-ns molecular dynamics (MD) simulation was conducted to explore the dynamic characteristics of the *CCNB2*–wogonin, *CCNB2*–kaempferol, and *CHEK1*–luteolin complexes obtained through molecular docking. The MD simulation outcomes, particularly those related to the root mean square deviation (MSD), root mean square fluctuation (RMSF), radius of gyration (Rg), solvent accessible surface area (SASA), and hydrogen bonds (H-bonds), are essential to assess the stability of both chemical compounds and protein complexes. Additionally, these simulations facilitate the evaluation of the stability of protein tertiary structures after binding with small molecules, the hydrophobicity of amino acid residues, and other pertinent factors.

#### 3.6.1 Molecular dynamics simulation of the *CCNB2–*wogonin complex

**Figure 6:**
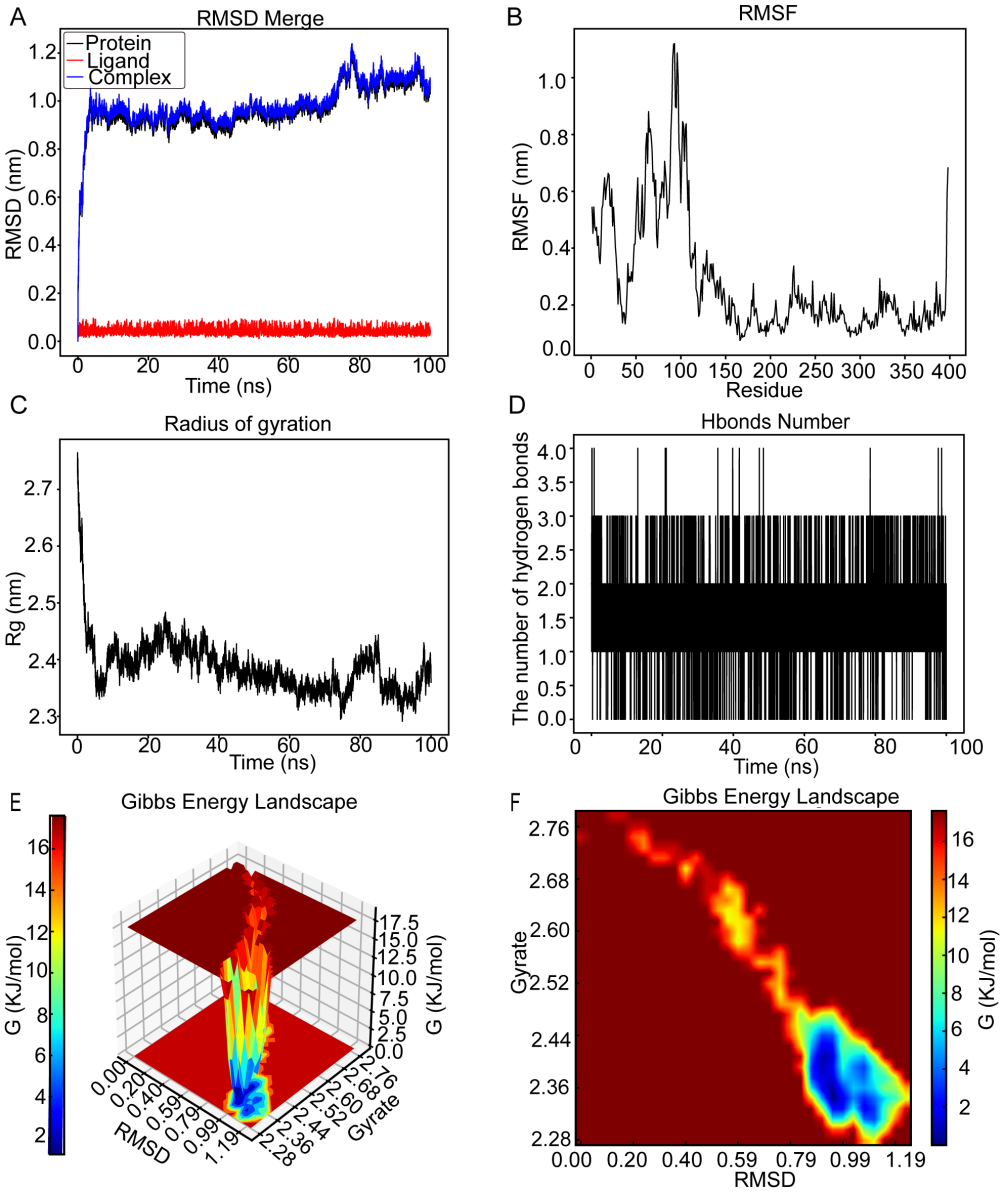
The molecular dynamics’ simulation of cyclin B2 (CCNB2)–wogonin complex over a 100-ns simulation. **(A)** Root mean square deviation (RMSD) plot showing the structural stability of the CCNB2 (black), wogonin (red), and CCNB2–wogonin complex (blue) over a 100-ns simulation. Stable RMSD values after ~20 ns suggest a well-equilibrated complex. **(B)** Root mean square fluctuation (RMSF) plot illustrating residue-level flexibility of CCNB2, with higher fluctuations observed at loop regions and termini. **(C)** Radius of gyration (Rg) analysis demonstrating the compactness of the protein throughout the simulation; stable Rg values (~2.4 nm) suggest preserved the tertiary structure. **(D)** Number of hydrogen bonds between CCNB2 and wogonin over time, indicating consistent binding interactions (1–3 H-bonds maintained). **(E)** 3D Gibbs free energy landscape plotted against RMSD and Rg, identifying energetically favorable conformational states. **(F)** 2D Gibbs energy projection mapping conformational energy basins, with deep blue areas representing the most stable binding conformations.

As illustrated in **Figure 6A**, the RMSD curve for wogonin (red line) remained stable at approximately 0.04 nm over the initial 100 ns. Meanwhile, the RMSD curve for *CCNB2* (black line) exhibited relative stability, ranging between 0.8 and 1 nm, from 2 to 75 ns. The RMSD curves of the *CCNB2*–wogonin complex (blue line) similarly showed stability, hovering around 0.8 to 1 nm from 2 to 74 ns, suggesting a stable interaction between wogonin and *CCNB2*. **Figure 6B** reveals that residues 0–9, 12–30, 40–110, and 111–120 of the *CCNB2*–wogonin complex exhibited greater flexibility. **Figure 6C** demonstrates that the Rg value of *CCNB2* remained consistent for 78 ns, with an average range of 2.31 to 2.49 nm. Hydrogen bonding, a potent non-covalent interaction, played a significant role in the stability of the *CCNB2*–wogonin complex, with a consistent presence of one to three stable hydrogen bonds throughout the MD simulation. Notably, the complex achieved a maximum of four hydrogen bonds during the simulation, as shown in **Figure 6D**. These hydrogen bonds between the ligand and receptor contributed to the stabilization of the complex. The 3D and 2D Gibbs energy landscapes of the *CCNB2*–wogonin complex are presented in **Figures 6E, F**, respectively. The complex exhibited low Gibbs free energy levels when the Rg value ranged from 2.44 to 2.28 nm, and the RMSD value ranged from 0.79 to 1.19 nm.

#### 3.6.2 Molecular dynamics simulation of the *CCNB2*–kaempferol complex

**Figure 7:**
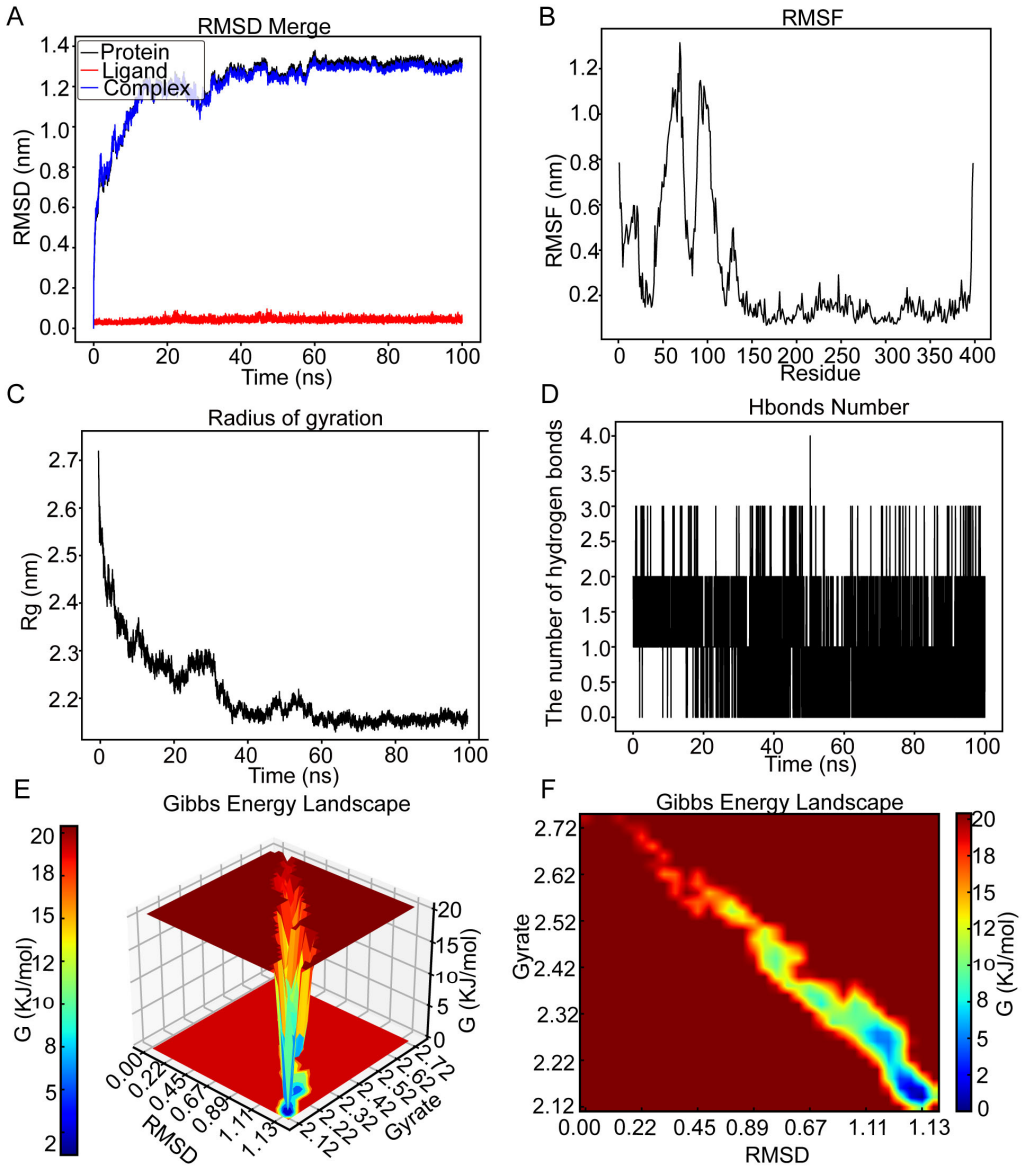
MD simulation analysis for the CCNB2–kaempferol complex over a 100-ns simulation. **(A)** RMSD plot showing the structural stability of the CCNB2 protein (red) and ligand RMSD of kaempferol (blue) over a 100-ns simulation. Stable RMSD values after ~30 ns suggest a well-equilibrated complex. **(B)** RMSF analysis of CCNB2 reveals low flexibility at the binding site and high flexibility at loop regions and termini. **(C)** The Rg of CCNB2 remains stable throughout the simulation, consistently around ~2.0 nm, indicating structural compactness. **(D)** Number of hydrogen bonds between CCNB2 and kaempferol over time. The complex consistently forms a few hydrogen bonds during the simulation. **(E)** 3D Gibbs free energy landscape plotted against RMSD and Rg, identifying energetically favorable conformational states. **(F)** 2D Gibbs energy projection mapping conformational energy basins, with deep blue areas representing the most stable binding conformations.

The RMSD graph for kaempferol (red line) stabilized at roughly 0.04 nm throughout the time frame of 0 to 100 ns, as shown in **Figure 7A**. Similarly, the RMSD graph for *CCNB2* (black line) exhibited relative stability, fluctuating between 1.08 and 1.27 nm from 10 to 28 ns before reaching a plateau at 1.3 nm from 65 to 100 ns. The RMSD graph for the *CCNB2*–kaempferol interaction (blue line) revealed stability at around 1.3 nm during the interval of 65 to 100 ns, suggesting a stable interaction between *CCNB2* and kaempferol. The flexibility of residues 1–26 and 31–120 in the *CCNB2*-kaempferol assembly is highlighted in **Figure 7B**. According to **Figure 7C**, the radius of Rg for *CCNB2* maintained levels between 2.18 and 2.24 nm from 38 to 100 ns. Throughout the 20- to 100-ns period, the *CCNB2*–kaempferol complex preserved stable hydrogen bonds, reaching a maximum of four hydrogen bonds, which are essential for the complex’s stability, as depicted in **Figure 7D**. **Figures 7E, F** display the 3D and 2D Gibbs energy landscapes of the *CCNB2–*kaempferol complex, respectively. The complex exhibited low levels of Gibbs free energy when the Rg value ranged from 2.12 to 2.27 nm and the RMSD value was between 1.11 and 1.13.

#### 3.6.3 Molecular dynamics simulation of the *CHEK1–*luteolin complex

**Figure 8:**
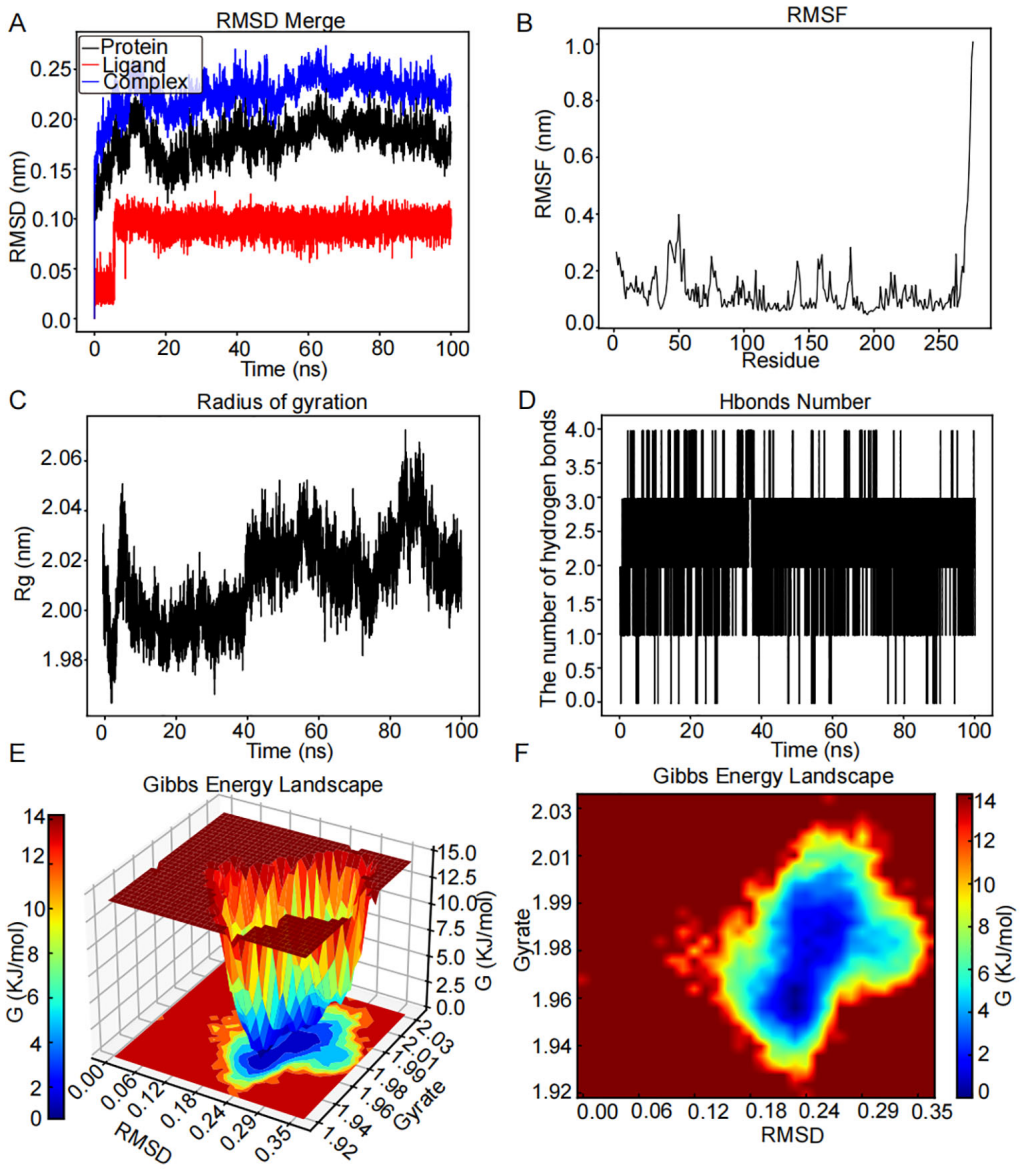
MD simulation analysis for the CHEK1–luteolin complex over a 100-ns simulation. **(A)** RMSD plot showing the structural stability of the CHEK1 protein (red) and ligand RMSD of luteolin (blue) over a 100-ns simulation. Stable RMSD values after ~10 ns suggest a well-equilibrated complex. **(B)** RMSF analysis of CHEK1 reveals that most residues fluctuate <0.15 nm, with higher peaks (~0.20–0.25 nm) at flexible loop regions. **(C)** The Rg of CHEK1 remains stable throughout the simulation, ranging consistently between 1.98 and 2.06 nm, indicating that the protein maintains its structural compactness. **(D)** Number of hydrogen bonds between CHEK1 and luteolin over time. The complex consistently forms a few hydrogen bonds during the simulation. **(E)** 3D Gibbs free energy landscape plotted against RMSD and Rg, identifying energetically favorable conformational states. **(F)** 2D Gibbs energy projection mapping conformational energy basins, with deep blue areas representing the most stable binding conformations.

The RMSD graph for luteolin (red line) remained stable within the range of 0.08 to 0.11 nm from 6 to 100 ns, as illustrated in **Figure 8A**. In a similar manner, the RMSD graph for *CHEK1* (black line) exhibited relative consistency, oscillating between 0.13 and 2.1 nm throughout the 20- to 100-ns period. The RMSD graph for the *CHEK1*–luteolin complex (blue line) suggested stability in the vicinity of 0.18 to 0.25 nm across the same timeframe, indicating a potential stable interaction between *CHEK1* and luteolin. Greater flexibility was observed in the residues 34–59, 62–71, 131–144, 154–169, and 178–186 within the *CHEK1*–luteolin complex, as demonstrated in **Figure 8B**. **Figure 8C** indicates that the radius of Rg of *CHEK1* remained within a range of 1.98 to 2.02 nm during the 5- to 40-ns interval. Throughout the molecular dynamics simulation, the *CHEK1*–luteolin complex maintained stable hydrogen bonds, varying between 1 to 3, with a maximum of four hydrogen bonds detected (**Figure 8D**). These hydrogen bonds are essential for the stability of the complex. The 3D and 2D Gibbs free energy landscapes of the *CHEK1*–luteolin complex are presented in **Figures 8E, F**, respectively. The complex displayed low Gibbs free energy levels when the Rg value was between 1.92 and 2.03 nm and the RMSD value varied from 0.06 to 0.35 nm.

### 3.7 Validation of FSL against HAdV *in vitro*

**Figure 9:**
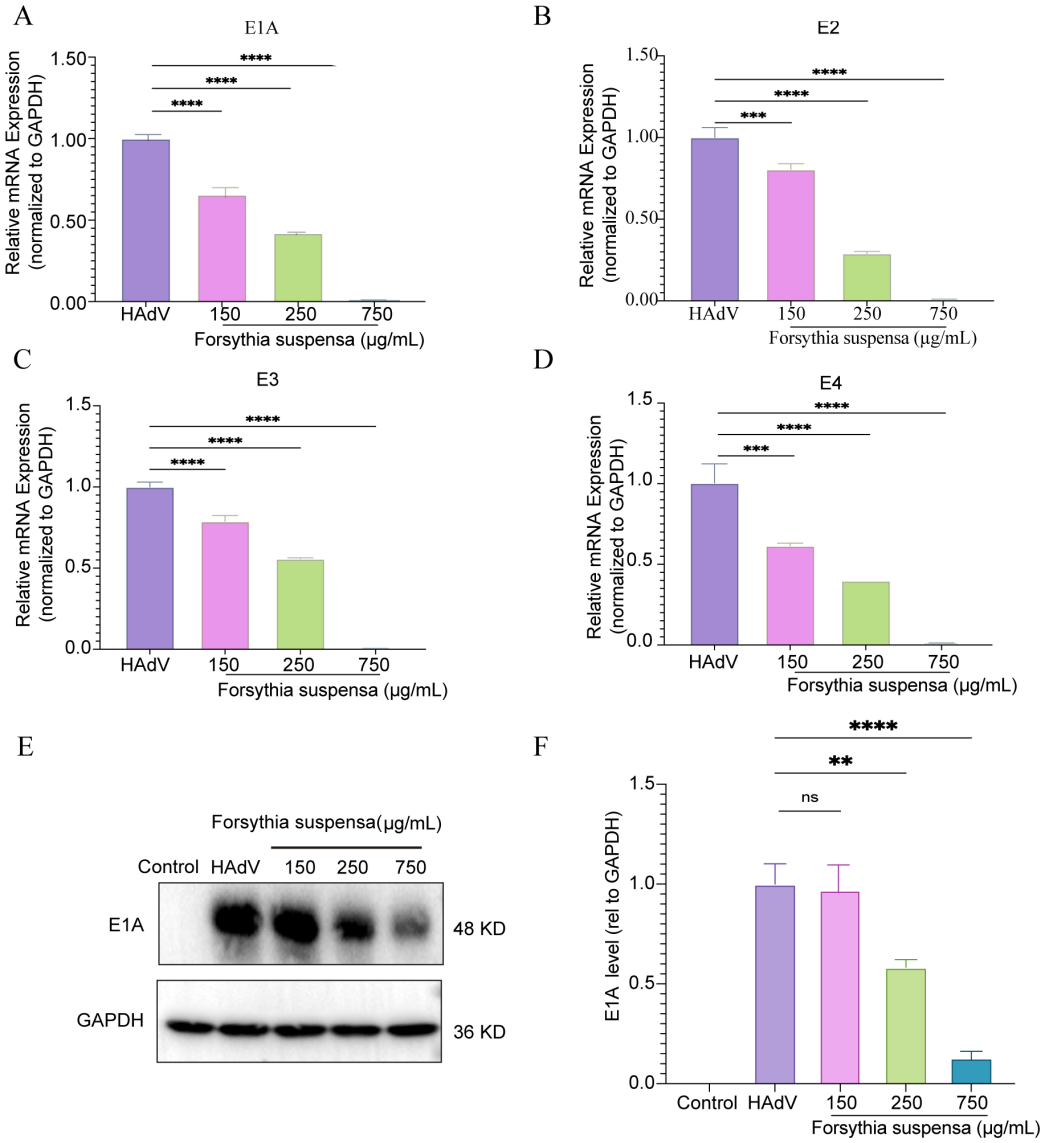
*In vitro* experiments validated the FSL inhibition of HAdV infection. A549 cell cultures were treated with varying concentrations of FSL prior to HAdV infection (MOI = 1) for 24 h. Real-time PCR and immunoblotting were used to detect HAdV protein or gene expression. **(A–D)** Real-time PCR was utilized to analyze the levels of the HAdV E1A, E2, E3, and E4 mRNA expression and normalized to GAPDH. **(E, F)** Immunoblotting was used to detect the expression of HAdV E1A proteins and GAPDH. The experimental procedures were independently replicated three times. The data are presented as mean ± SD (*n* = 3) of one experiment. Statistical significance was denoted as ns, no significant, ****p<0.00001, **p < 0.01, and ***p < 0.0001.

Adenovirus is a double-stranded DNA virus that includes early genes (*E1A*, *E1B*, *E2*, *E3*, and *E4*) and late genes (*L1*, *L2*, *L3*, *L4*, and *L5*). To confirm the impact of FSL treatment for HAdV infection, A549 cells were treated with FSL to assess the expression of HAdV early genes (*E1A*, *E2*, *E3*, and *E4*) and *E1A* proteins. FSL concentrations (150–750 μg/mL) were tested via CCK-8 assay and >90% cell viability was confirmed in all samples (**Supplementary Figure S1**). A549 cells were exposed to 24 h of varying different concentrations of FSL (150–750 μg/mL) prior to 2 h of HAdV infection. The expression of the *E1A*, *E2*, *E3*, and *E4* genes was examined using real-time PCR. FSL led to a reduction in HAdV gene expression in a dose-dependent manner compared to that in the control group (**Figures 9A–D**). FSL also reduced the expression of HAdV *E1A* protein in a manner that depended on the dose when compared to the control group (**Figures 9E, F**). These results imply that FSL may be effective in treating HAdV infections.

### 3.8 FSL has significant G2/M arrest effect during HAdV infection

**Figure 10:**
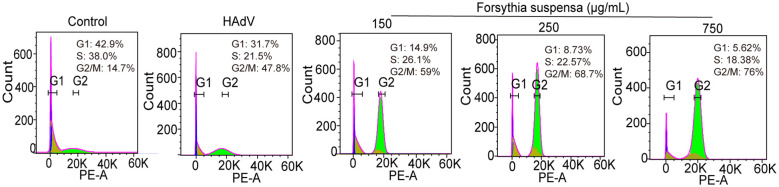
Cell cycle effects of FSL on A549 cells during HAdV infection. A549 cell cultures were pre-treated with varying concentrations of FSL prior to HAdV infection (MOI = 1) for 24 h. Flow cytometry analysis was conducted to assess the impact on cell cycle progression. The percentage distribution of cells in the G1, S, and G2 phases is indicated for each treatment condition. The experimental procedures were independently replicated three times.

To evaluate the impact of FSL on HAdV-induced cell cycle alterations, A549 cells or BEAS-2B cells were pretreated with escalating FSL concentrations for 24 h prior to infection with HAdV (MOI = 1). Flow cytometric analysis revealed a dose-dependent shift in cell cycle distribution. Compared with untreated controls, FSL-exposed A549 cells exhibited significantly reduced G1-phase populations (from 31.7% to 5.62%) with concomitant G2/M-phase accumulation (increasing from 47.8% to 76%) (**Figure 10**). Moreover, we found that FSL-exposed BEAS-2B cells increased the G2-phase population (**Supplementary Figure S2**). The distinct G2/M phase arrest pattern suggests that FSL may disrupt normal cell cycle progression during HAdV infection.

### 3.9 Validation of the role of hub genes in the modulation of HAdV by FSL

**Figure 11:**
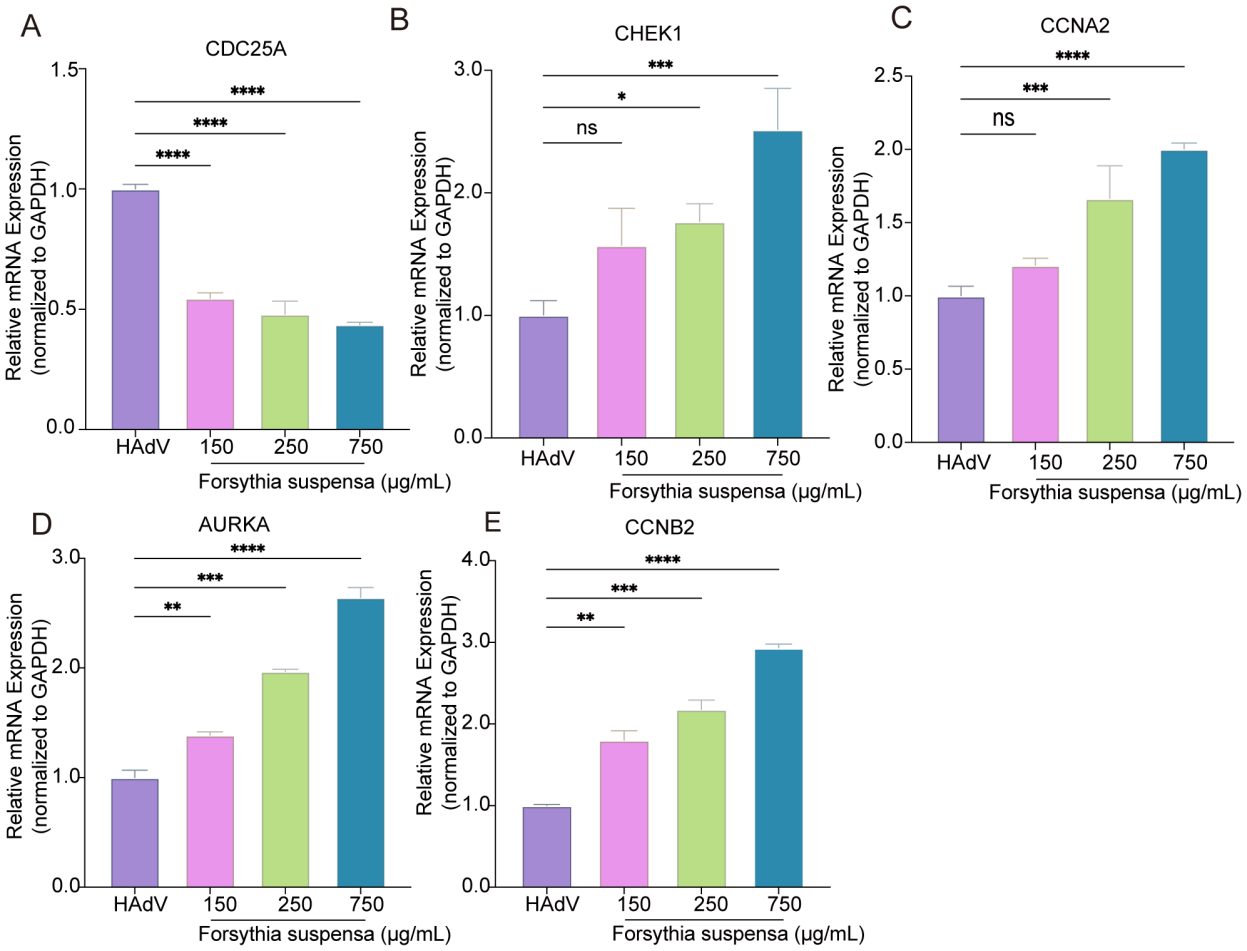
Validation of cell cycle-related gene in the regulation of FSL for HAdV. **(A–E)** A549 cell cultures were treated with varying concentrations of FSL prior to HAdV infection (MOI = 1) for 24 h. Real-time PCR was utilized to analyze the expression levels of the *CDC25A*, *CHEK1, CCNA2*, *AURAK*, and *CCNB2* genes and normalized to GAPDH. The experimental procedures were independently replicated three times. The data are presented as mean ± SD (*n* = 3) of one experiment. Statistical significance was denoted as *p < 0.05, **p < 0.01, ***p < 0.001, ****p < 0.0001.

To evaluate the effect of FSL treatment on the expression of hub genes during HAdV infection, A549 cells were pretreated with FSL before being infected with HAdV, allowing for the assessment of hub gene expression. Following 24 h of pretreatment with different concentrations of FSL prior to HAdV infection, qRT-PCR and Western blot analyses revealed distinct regulatory effects on cell cycle-related targets. Compared to controls, FSL treatment significantly downregulated the expression of the hub gene *CDC25A* while dose-dependently increasing the mRNA levels of *CCNA2*, *AURKA*, *CHEK1*, and *CCNB2* (**Figure 11**). Consistent with the qRT-PCR results, the CDC25A protein levels decreased significantly, whereas the *CCNB2*, *CCNA2*, *CHEK1*, and *AURKA* levels were markedly increased following FSL treatment (**Supplementary Figure S3**). These findings suggest that FSL treatment may influence the hub genes associated with HAdV infection.

## 4 Discussion

Human adenovirus (HAdV) infections manifest as diverse clinical manifestations ranging from respiratory illnesses and gastroenteritis to hepatitis. With high contagiousness and significant mortality risks in immunocompromised populations, these infections currently lack FDA-approved antiviral therapies. Traditional Chinese medicine (TCM) has emerged as a promising alternative due to its unique therapeutic efficacy and favorable safety profile. Notably, FSL, a TCM formulation with documented “heat-clearing” and “detoxifying” properties, has shown empirical effectiveness against HAdV infections. However, its precise antiviral mechanisms remain elusive. This study elucidates FSL’s novel mechanism in mitigating HAdV infection through cell cycle regulation via hub gene modulation, with particular emphasis on the functional contributions of its bioactive flavonoid components. Multi-omics integration revealed 39 bioactive components, especially flavonoids, targeting cell cycle regulators, with molecular simulations confirming interactions of flavonoid-crucial regulators like *CDC25A*, *CCNB2*, *AURKA*, *CHEK1*, and *CCNA2*. Functionally, FSL suppressed viral replication via G2/M arrest by downregulation of *CDC25A* expression and upregulation of *CCNB2*, *AURKA*, *CHEK1*, and *CCNA2* expression. Our findings bridge empirical dietary practices with precision antiviral strategies, offering a TCM for combating cell cycle-dependent viral pathogens through phytochemical synergism.

We firstly identified 39 key active ingredients of FSL by UPLC/Q-TOF MS, including four flavonoid compounds: kaempferol, luteolin, quercetin, and wogonin, all of which demonstrate antiviral activity (Zumerle et al., 2024). Luteolin, a significant natural flavonoid, possesses various essential biological properties such as anti-inflammatory, immune-modulating, and antitumor activities. It can be found in a range of vegetables, fruits, and several herbs used in traditional medicine (Lu et al., 2023). Luteolin has been shown to exhibit antiviral effects. It limits the replication of the dengue virus through the inhibition of the host enzyme furin (Peng et al., 2017). This enzyme plays a vital role in the processing of the pr moiety from the prM protein of immature virus particles within the trans-Golgi network, which is essential for the maturation of virions throughout the viral life cycle. Additionally, luteolin mitigates the inflammatory response induced by viruses in RAW264.7 cells by blocking STAT1/3-dependent NF-κB signaling and facilitating the activation of HO-1 (Liu et al., 2016). Luteolin suppresses the replication of respiratory syncytial virus by modulating the *MiR-155*/*SOCS1*/*STAT1* signaling pathway (Wang et al., 2020). Quercetin, a type of flavonoid often present in various fruits and vegetables (Anand David et al., 2016), is known for its properties as an antioxidant, antiviral agent, antimicrobial compound, and anti-inflammatory substance (Di Petrillo et al., 2022). Several studies have reported that quercetin may serve as an antiviral agent, mainly by blocking the initial phases of viral infection, engaging with proteases crucial for viral replication, and alleviating inflammation resulting from infection (Di Pierro et al., 2021). Quercetin has been shown to possess antiviral properties against multiple viruses, including SARS-CoV-2 (Yang et al., 2023), Zika virus (Saivish et al., 2023), influenza A virus (IAV) (Luo et al., 2023), and hepatitis C virus (HCV) (Khachatoorian et al., 2012). Wogonin is a natural flavonoid derived from *Scutellaria baicalensis*, which is known for its diverse pharmacological effects, including anti-cancer, antiviral, anti-inflammatory, and immune-modulating activities (Banik et al., 2022; Zhao et al., 2022).Wogonin exhibits antiviral activity against hepatitis B virus (HBV) both *in vitro* and *in vivo* (Guo et al., 2007). Wogonin, a natural flavonoid, exhibits antiviral effects against influenza by regulating the AMPK pathways (Seong et al., 2018). Kaempferol and its derivatives are recognized for their antioxidant, anti-inflammatory, and antimicrobial effects, among other health benefits (Periferakis et al., 2023). Kaempferol demonstrates antiviral activity against the African swine fever virus (Arabyan et al., 2021), hepatitis B virus (Rashed et al., 2014), human cytomegalovirus (HCMV) (Mitrocotsa et al., 2000), and pseudorabies virus (Chen et al., 2021). FSL has a molecular basis for the treatment of HAdV infection and can prevent and treat HAdV infection through the joint action of multiple targets.

The GO analysis of 30 common targets revealed that FSL is involved in G2 to M phase. The KEGG signaling pathway analysis of the therapeutic targets also indicated their involvement in the cell cycle. We also found four core targets (*CCNA2*, *CDC25A*, *CHEK1*, and *CCNB2* related to the cell cycle process) to be involved. Furthermore, the evaluation of the key targets alongside the active components obtained was performed through molecular docking, featuring components such as luteolin, kaempferol, quercetin, and wogonin. The docking results revealed a binding affinity that varied between –6.7 and -9.2 kJ/mol. The flavonoid components exhibited a significant binding activity toward the related cell cycle targets. Furthermore, molecular dynamics simulations revealed that flavonoid components exhibit preferential binding affinity toward core regulators of the cell cycle, with the results demonstrating robust complex stability through dynamic interaction analyses. These results showed the potential role of these active components in enhancing the therapeutic impact of FSL on HAdV.

FSL inhibited viral early gene and protein expression by modulating the cell cycle progression, specifically inhibiting *CDC25A* and promoting *CCNB2*, *AURKA*, *CCNA2*, and *CHEK1* expression. *CHEK1* increase and *CDC25A* degradation may activate checkpoints. The coordinated upregulation of *CCNB2* and *CCNA2* may influence paradoxical *CDK1/2* inactivation. *AURKA* kinase inhibition inducing spindle assembly defects disrupt mitosis. *AURKA* was involved in regulating the G2/M cell cycle and associated with EBV virus infection (Varshney et al., 2023). Viral infection, viral protein expression, or the presence of viral DNA can cause host cell cycle arrest during the G2/M phase (Davy and Doorbar, 2007). The arrest at the G2/M phase induced by the virus might influence the amounts of proteins within the cell, managing the gene expression required for the viral life cycle to progress. This G2/M arrest could push host cells into a pseudo-S phase state, enabling HAdV to take advantage of cellular replication resources and processes, thereby prolonging the replication duration to facilitate ongoing replication and increase the quantity of viral genes. Focusing on the cell cycle could improve adenoviral replication that is independent of *E1A* (Ehrenfeld et al., 2023). Typically, the *E1A* protein of HAdV facilitates the progression of the cell cycle in cells that have been infected, resulting in an environment that is supportive of viral replication. However, employing strategies that target the cell cycle, such as inhibiting G1/S phase or G2/M phase transitions, can effectively reduce the reliance on *E1A* mechanisms, allowing HAdV to replicate smoothly even in the absence of *E1A*. An HAdV early region 4 deletion mutant causes G2/M phase arrest through the activation of Ataxia Telangiectasia Mutated (*ATM*) and decreases the expression of the mitotic marker phosphorylated (ser10) histone 3 (Kafle et al., 2022). Therefore, FSL inhibited HAdV infection by regulating DNA replication during the G2/M phase.

Despite providing comprehensive *in vitro* and *in silico* evidence that FSL flavonoids interfere with cell cycle regulators to inhibit HAdV replication, our study has several important limitations. First, we have not yet deconvoluted individual flavonoids or non-flavonoids nor fully characterized any synergistic interactions or fully characterized potential synergistic interactions. Second, while molecular docking and 100-ns MD simulations suggest stable flavonoid–protein binding, direct structural validation (e.g., X-ray crystallography, SAXS, or SPR) has not been performed. Third, we lack *in vivo* experiments to validate that FSL flavonoids inhibited HAdV replication via targeting to cell cycle regulators. In a future work, we will undertake additional studies to address these limitations, thereby bridging our mechanistic insights toward therapeutic development.

## 5 Conclusion

Integrating phytochemical profiling with multi-omics exploration, this study systematically deciphered the anti-adenoviral mechanism of FSL. A total of 39 UPLC/Q-TOF-MS-characterized bioactive constituents synergistically targeted 556 predicted targets, with network pharmacology identifying 30 functional intersections between FSL targets and HAdV-associated DEGs. The core regulators *CCNB2* and *CHEK1* emerged as pivotal hubs through the PPI network topology analysis. Mechanistically, FSL extracts induced G2/M arrest via the downregulation of *CDC25A* expression and upregulation of *CCNB2*, *AURKA*, *CHEK1*, and *CCNA2* expression. These findings not only validate FSL’s traditional medicinal applications but also provide a paradigm to develop plant-derived multi-target antivirals, particularly against DNA viruses exploiting host cell cycle progression.

## Data Availability

The original contributions presented in the study are included in the article/**Supplementary Material**. Further inquiries can be directed to the corresponding author.
