# Supplementary material for: Flavonoid compound from Forsythia suspensa leaves inhibits adenovirus infection related to cell cycle based on UHPLC-Q-Exactive-Orbitrap/MS and experimental validation
Source: Front Cell Infect Microbiol. 2025 Aug 21;15:1627863. doi: 10.3389/fcimb.2025.1627863 (PMC12408589; doi:10.3389/fcimb.2025.1627863)
Supplement: Supplementary file 1 [file DataSheet1.docx]

**Supplementary Material:**

Figure S1: Cytotoxicity measurement. Monolayers of A549 cells in triplicate wells were treated with a test compound at 150, 250 or 750 μg/mL concentrations for 48 h, cytotoxic effect was determined colorimetrically using MTT (n=3). Shown are the means ± SD. ** p ≤ 0.01. The experiments were performed independently 3 times.


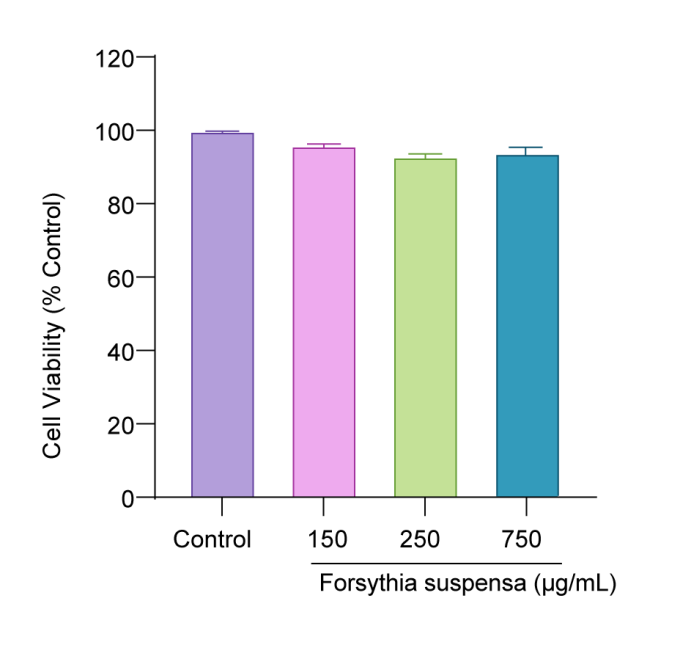


Figure S2. In vitro experiments validated FSL inhibition of HAdV infection arrests BEAS-2B cells at G2/M. BEAS_2B cells cultures were treated with varying concentrations of FSL (150-750 μg/mL) prior HAdV infection (MOI=1) for 24 h, real-time PCR and immunoboting were used to detected HAdV protein or gene expression. Flow cytometry analysis was conducted to assess the impact on cell cycle progression. A. Cytotoxicity measurement. Monolayers of BEAS-2B in triplicate wells were treated with a test compound at 150, 250 or 750 μg/mL concentrations for 48 h, cytotoxic effect was determined colorimetrically using MTT (n=3). B.Immunoblotting was used to detect the expression of HAdV E1A proteins and GAPDH. (C)Real-time PCR was utilized to analyze the expression levels of the HAdV E1A, E2, E3, E4 mRNA expression and normalized to GAPDH. (D)Cell cycle effects of FSL on BEAS-2B Cells during HAdV infection. The percentage distribution of cells in the G1, S, and G2 phases is indicated for each treatment condition. The experimental procedures were independently replicated three times. The data are presented as mean±SD (n=3) of there experiment. Statistical significance was denoted as *p < 0.05, **p < 0.01, and ***p < 0.0001.


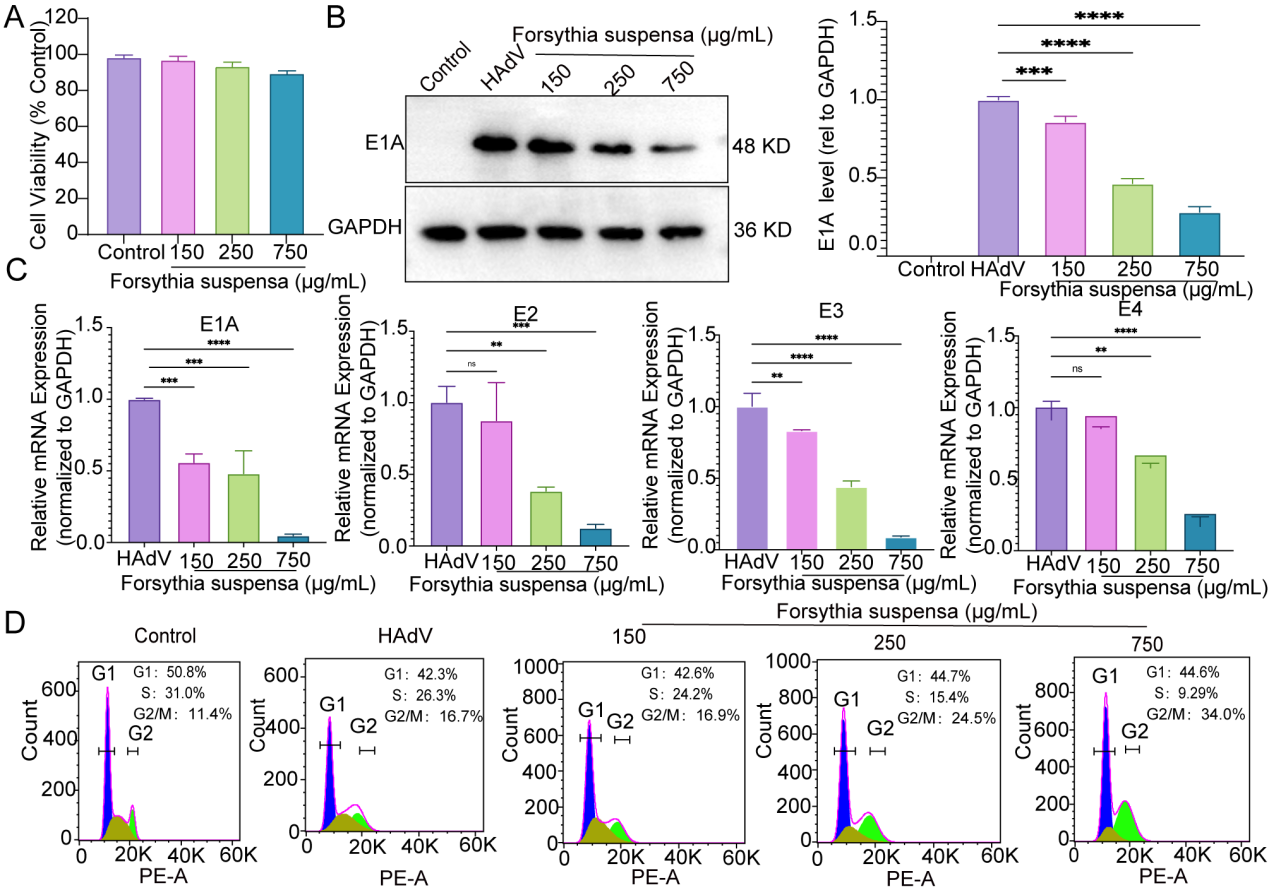


Figure S3. Validation of cell cycle-related protein expression level in the regulation of FSL for HAdV by Western blot. A549 cell cultures were treated with varying concentrations of FSL prior HAdV infection (MOI=1) for 24 h. Western blot was utilized to analyze the expression levels of the CDC25A, CHEK1, CCNA2, AURAK, and CCNB2 protein and normalized to GAPDH. The experimental procedures were independently replicated three times.


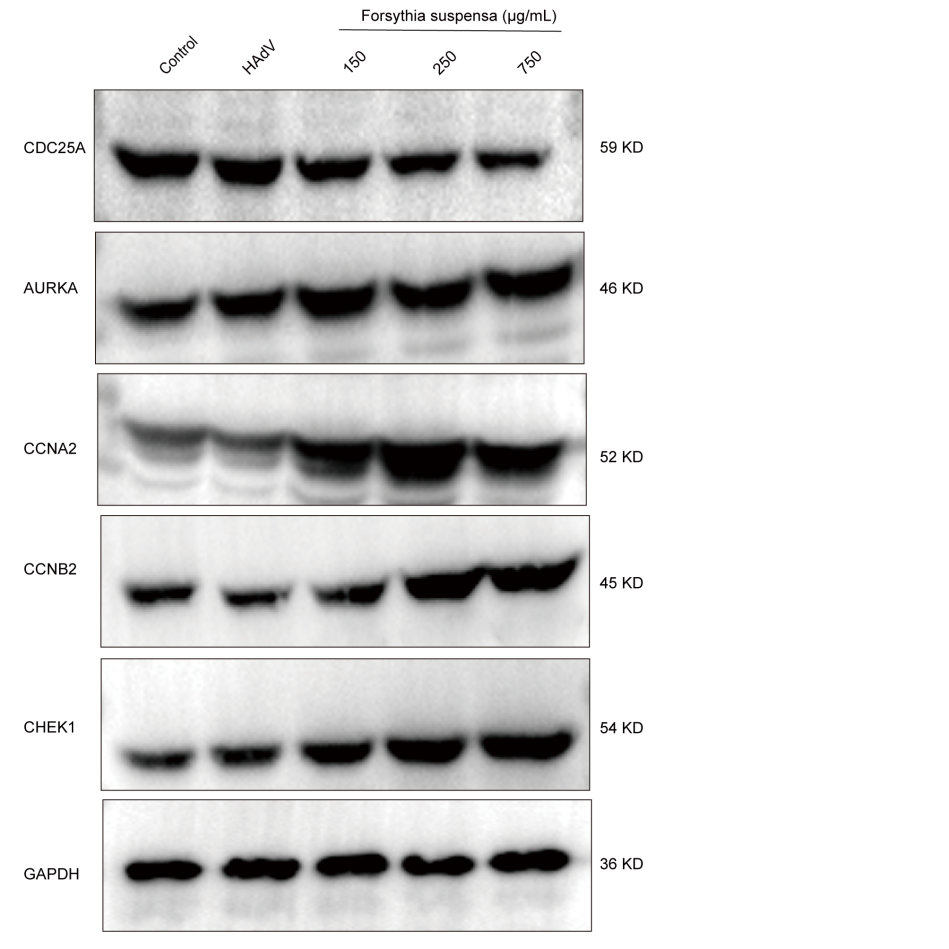


Figure S4: Raw data of uncropped full Western blot bands.


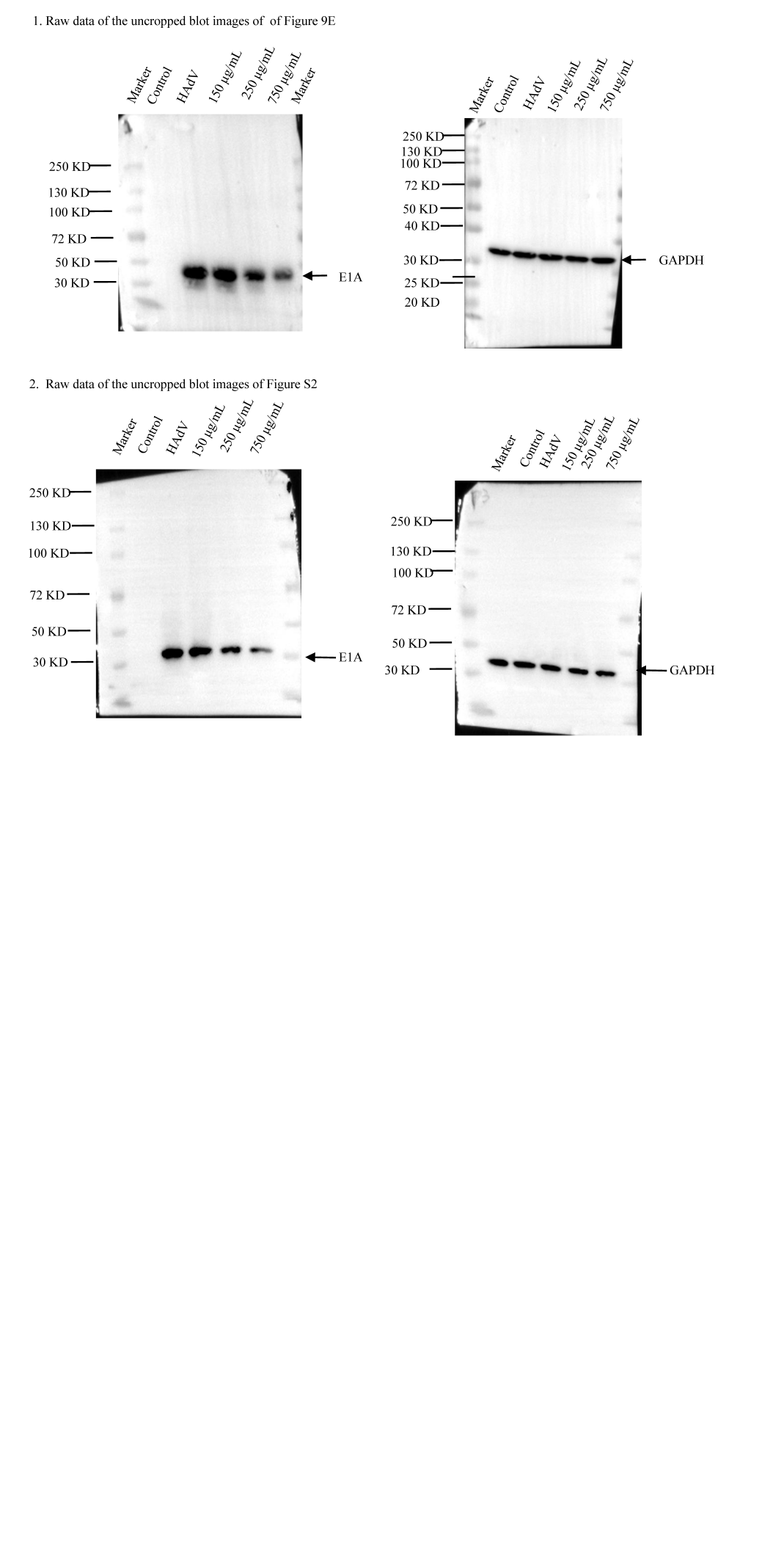


Table S1. Raw qPCR Ct Values for HAdV Gene Expression in the A549 Cell Infection Model

| Group  Gene | control | | | HAdV | | | 150 µg/mL FSL | | | 250 µg/mL | | | 750 µg/mL | | |
| --- | --- | --- | --- | --- | --- | --- | --- | --- | --- | --- | --- | --- | --- | --- | --- |
|  | n=1 | n=2 | n=3 | n=1 | n=2 | n=3 | n=1 | n=2 | n=3 | n=1 | n=2 | n=3 | n=1 | n=2 | n=3 |
| GAPDH | 16.514 | 16.498 | 16.489 | 15.989 | 16.054 | 15.782 | 16.176 | 16.359 | 16.194 | 16.320 | 16.403 | 16.161 | 17.399 | 17.364 | 17.369 |
| E1A | 26.691 | 26.535 | 26.872 | 19.985 | 19.977 | 20.068 | 20.994 | 20.736 | 20.876 | 21.623 | 21.548 | 21.632 | 24.623 | 24.694 | 24.700 |
| E2 | 26.432 | 26.375 | 26.557 | 21.051 | 21.043 | 21.197 | 21.589 | 21.702 | 21.682 | 23.290 | 23.175 | 23.200 | 27.361 | 27.294 | 27.592 |
| E3 | 30.879 | 30.978 | 30.992 | 19.230 | 19.160 | 19.152 | 19.785 | 19.825 | 19.700 | 20.375 | 20.346 | 20.370 | 25.245 | 25.358 | 25.185 |
| E4 | 28.311 | 28.501 | 28.272 | 23.437 | 23.522 | 23.774 | 24.483 | 24.558 | 24.536 | 25.251 | 25.137 | 25.351 | 27.329 | 27.225 | 27.158 |

Table S2. Raw qPCR Ct Values for Cell cycle Gene Expression in the A549 Cell Infection Model

| Group  Gene | control | | | HAdV | | | 150 µg/mL FSL | | | 250 µg/mL | | | 750 µg/mL | | |
| --- | --- | --- | --- | --- | --- | --- | --- | --- | --- | --- | --- | --- | --- | --- | --- |
|  | n=1 | n=2 | n=3 | n=1 | n=2 | n=3 | n=1 | n=2 | n=3 | n=1 | n=2 | n=3 | n=1 | n=2 | n=3 |
| GAPDH | 17.236 | 17.583 | 17.245 | 19.198 | 19.184 | 19.185 | 16.617 | 16.652 | 16.612 | 16.471 | 16.574 | 16.487 | 17.011 | 17.028 | 17.021 |
| CDC25A | 29.332 | 29.513 | 29.370 | 29.180 | 29.210 | 29.235 | 27.509 | 27.594 | 27.477 | 27.752 | 27.431 | 27.638 | 28.251 | 28.199 | 28.249 |
| CHECK1 | 25.914 | 25.872 | 25.999 | 27.760 | 27.971 | 28.085 | 24.519 | 24.650 | 25.076 | 24.417 | 24.354 | 24.587 | 24.337 | 24.320 | 24.674 |
| CCNA2 | 24.127 | 24.050 | 23.900 | 26.260 | 26.079 | 26.195 | 23.353 | 23.404 | 23.290 | 22.798 | 22.583 | 22.964 | 23.036 | 22.981 | 22.998 |
| ARUKA | 21.678 | 21.649 | 21.657 | 24.513 | 24.378 | 24.501 | 21.396 | 21.435 | 21.412 | 20.810 | 20.790 | 20.814 | 20.907 | 20.837 | 20.912 |
| CCNB2 | 23.238 | 23.013 | 21.124 | 25.773 | 25.746 | 25.753 | 22.421 | 22.290 | 22.319 | 21.915 | 22.021 | 21.934 | 22.050 | 22.020 | 22.030 |
